# *Viscum album* mother tinctures: Harvest conditions and host trees influence the plant metabolome and the glycolytic pathway of breast cancer cells

**DOI:** 10.3389/fphar.2022.1027931

**Published:** 2022-10-31

**Authors:** Michelle Nonato de Oliveira Melo, Alan Clavelland Ochioni, Patricia Zancan, Adriana Passos Oliveira, Mirio Grazi, Rafael Garrett, Carla Holandino, Stephan Baumgartner

**Affiliations:** ^1^ Multidisciplinary Laboratory of Pharmaceutical Sciences, Faculty of Pharmacy, Universidade Federal do Rio de Janeiro, Rio de Janeiro, Brazil; ^2^ Metabolomics Laboratory, Chemistry Institute, Universidade Federal do Rio de Janeiro, Rio de Janeiro, Brazil; ^3^ Laboratório de Oncobiologia Molecular (LabOMol), Faculty of Pharmacy, Universidade Federal do Rio de Janeiro, Rio de Janeiro, Brazil; ^4^ Hiscia Institute, Society for Cancer Research, Arlesheim, Switzerland; ^5^ Institute of Integrative Medicine, University of Witten/Herdecke, Herdecke, Germany; ^6^ Institute of Complementary and Integrative Medicine, University of Bern, Bern, Switzerland

**Keywords:** *Viscum album*, mistletoe, metabolome, multivariate analysis, glycolytic enzymes, anticancer, *in vitro*

## Abstract

*Viscum album* is a semi-parasitic plant used for over one hundred years in complementary cancer therapy. The main commercial drugs used in cancer patients’ treatment are derived from the aqueous *V. album* extracts, whose cytotoxic potential is mostly attributed to the aqueous soluble antitumoral metabolites. On the counterpart, ethanol solvents must be used to obtain *V. album* mother tinctures. This methodology permits better solubilization of phenolic compounds, among others, which present antitumoral bioactivity. Recently, the metabolomics approach revealed the influence of the host tree on the *V. album* subspecies differentiation. To increase the scientific information about the chemical differences related to the host trees and to clarify the seasonal influences, in this study, the metabolome of 50 *V. album* mother tinctures from three subspecies (*abietis*, *album*, *austriacum*) and five host trees (*Malus domestica*, *Quercus sp.*, *Ulmus carpinifolia*, *Pinus sylvestris*, *Abies alba*) was evaluated using summer and winter plant harvests. The *in vitro* cytotoxic activities were investigated in breast cancer cells (MDA-MB-231) and immortalized normal human keratinocytes (HaCaT). The summer *V. album* mother tinctures presented higher cytotoxic activity than winter ones. Among the summer samples, those prepared with *V. album* subsp. *album* were more cytotoxic than *V. album* subsp. *abietis* and subsp. *V. album* subsp. *austriacum*. The *V. album* harvested from *Quercus petraea* and *Abies alba* inhibited the key-glycolytic enzymes: hexokinase (HK), phosphofructokinase (PFK), pyruvate kinase (PK). This activity was related to a reduction in glucose uptake and lactate production, which were host-tree-time-dose-dependent. The untargeted metabolomic approach was able to discriminate the mother tinctures according to respective botanical classes and harvest season. A total of 188 metabolites were annotated under positive and negative modes. Fourteen compounds were responsible for the samples differentiation, and, to the best of our knowledge, eight were described in the *Viscum album* species for the first time. Our study shows the interruption of the Warburg effect as a novel antitumoral mechanism triggered by *V. album* mother tinctures, which is related to their metabolite profile. These results bring scientific evidence that encourages the use of *V. album* mother tinctures as a natural product for cancer therapy.

## 1 Introduction

Plants are an important source of medicinal compounds that have been used in the treatment of different diseases for a hundred years. Many plant-derived compounds with anti-cancer activity were discovered and were supported by evidence and use in clinical trials. However, it is known that beneficial effects on health, sometimes, lie in complex plant blend compounds, and not just in a specific molecule (Newman and Cragg, 2016).

*Viscum album* is a semi-parasitic plant that belongs to the Equisetopsida class, subclass Magnoliidae, superorder Santalanae, order Santalales, family Santalaceae and genus Viscum (Tropicos, 2022). The Santalaceae family has 39 genera, including *Viscum*, which has 49 species (The World Flora, 2022). *V. album* L. is mainly distributed in central and southwestern Europe and eastern Asia. Popularly known as Mistletoe, Mistel, or Gui in Europe, its distribution to the population in this continent is mainly divided into three subspecies, which have different host trees: *Viscum album* subsp. *album*, which grows on hardwood trees such as *Malus domestica* (apple tree), *Quercus* sp. (oak), and *Ulmus* sp. (elm); *Viscum album* subsp. *abietis* that grows on *Abies* sp. (conifers) and *Viscum album* subsp. *austriacum* that grows on *Pinus* sp. (conifers), the latter being softwood hosts (Becker, 2000).

Mistletoe was described in European folk medicine as a form of the treatment of epilepsy, liver diseases, ulcers, gout, and hypertension, for many years (Büssing, 2000; Lev et al., 2011). The treatment with mistletoe extracts represents an important oncological intervention due to its cytotoxic and immunomodulatory action (Tröger et al., 2013; Ostermann et al., 2020). Several *in vitro* and *in vivo* studies highlight the contribution of the following compounds to the antitumor effects of crude, aqueous, and fermented *V. album* extracts: 1) lectins, the most important being mistletoe viscolectins (proteins of high molecular weight, about 30 kDa); 2) viscotoxins (low molecular weight proteins, about 5 kDa) and 3) polysaccharides (Janssen et al., 1993; Büssing et al., 1996; Hajtó et al., 2005). The anticancer effect attributed to mistletoe aqueous extracts is based on two main mechanisms: direct cytotoxicity and stimulation of the immunomodulatory system. However, the *in vitro* cytotoxicity of *V. album* extracts is not only associated with the presence of viscolectins and viscotoxins, since non-aqueous preparations have demonstrated anticancer activity, which suggests the involvement of other compounds (Pietrzak et al., 2017; Melo et al., 2018; Zelovitis et al., 2019). Biologically active compounds such as phenolic acids, alkaloids, flavonoids, and phenylpropanoids seem to act synergistically, enhancing the antitumor effect of *V. album* extracts (Gómez et al., 1997; Yang et al., 2009; Kim et al., 2016; Melo et al., 2018).

On the other hand, different preparations of this species have been widely used in folk medicine in Europe, especially mother tinctures (MT), which are hydroalcoholic solutions of herbal drugs (Anvisa, 2011). The main use of the *V. album* tinctures is linked to blood pressure reduction, with dosages that vary according to different studies (European Medicines, 2011). Despite this traditional use of *V. album* tincture for hypertension treatment, studies have shown cytotoxic activity of these extracts for cancer assays. Stan et al. (2013) described the cytotoxic activity of the *V. album* hydroalcoholic extract to Erlich tumors in association with doxorubicin. This combination promoted greater control of tumor volume and reduction of cellular growth in treated animals. In this sense, Korga et al. (2019) showed that natural compounds, such as apigenin and hesperidin, produced an increase in the toxicity of doxorubicin in hepatocarcinoma cell line HepG2. Both flavonoids changed the expression of the two glycolytic pathway genes (HK2 and LDHA), which play a key role in the aerobic glycolysis of these cells.

It is known that cancer cells have a different metabolism to promote growth, survival, proliferation, and long-term maintenance. The increased glucose uptake and conversion of glucose to lactate is the most common feature of cancer metabolism. The conversion of glucose into lactic acid in the presence of oxygen is known as aerobic glycolysis or the Warburg effect, and this characteristic is only detected in cancer cells (Liberti and Locasale, 2016; Pascale et al., 2020; Bazylianska et al., 2021; Kalezic et al., 2021).

Regarding the promising anticancer activity of *V. album* mother tinctures, and the lack of evidence about their action in the glycolytic metabolism, this study evaluated the anticancer activity of these alcoholic preparations using MTT assay and enzyme kinetics of the hexokinase, phosphofructokinase, pyruvate kinase and glicose-6-phosphate dehydrogenase in the human breast cancer cell line MDA-MB-231. Additionally, liquid chromatography coupled with high-resolution mass spectrometry, followed by a multivariate statistical analysis was applied to show the influence of seasonal variations and the host-tree dependence on the biological activities investigated.

## 2 Materials and methods

### 2.1 Plant material

The plant material was taxonomically authenticated at the Universidade Estadual do Rio de Janeiro, Brazil. Fifty mother tinctures (MT) were prepared using three subspecies of *V. album* (*album*, *austriacum* and *abietis*) collected in Switzerland, in September 2017 (summer MT) and in January 2018 (winter MT). For each season, *V. album* (whole plant) from the following host trees were harvested in quintuplicate: *Malus domestica*, *Quercus robur* and *Quercus petrae*, *Ulmus carpinifolia, Abies alba*, *Pinus sylvestris*. To evaluate possible variations in MT chemical composition due to geographical and soil characteristics, samples were collected from different locations in Switzerland (geographic coordinates 47.471351, 7.692720), named: Disli, Höffli, Rütti, Rösli, Seewen, Courgenay, St. Pantaleon, Ermitage, Hoffmann, Widar and Hiscia. All harvests followed the same pattern as previously described (Jäger et al., 2021).

### 2.2 Mother tinctures preparation

*V. album* MT was prepared from fresh plant material according to previous methodology described by our group (Anvisa, 2011; Holandino et al., 2020). The fresh material was fragmented into parts smaller than 5 cm and submitted to an ethanolic maceration process for 21 days at room temperature shaking twice a day. The final alcoholic content ranged from 40 to 50% v/v. The mother tinctures were coded according to the respective host tree and the harvest season, as following: *Viscum album* subsp. *abietis* from *Abies alba* in summer (A-S) or winter (A-W), *Viscum album* subsp. *album* from *Malus domestica* in summer (M-S) or winter (M-W), *Viscum album* subsp. *album* from *Quercus* sp. in summer (Q-S) or winter (Q-W), *Viscum album* subsp. *album* from *Ulmus carpinifolia* in summer (U-S) or winter (U-W), *Viscum album* subsp. *austriacum* from *Pinus sylvestris* in summer (P-S) or winter (P-W).

### 2.3 Cell cultivation

MDA-MB-231 (triple negative human breast cancer cell line) was donated by the Laboratory of Cell Biology Applied to Medicine, Hospital Universitário Clementino Fraga Filho (UFRJ, Rio de Janeiro, Brazil). HaCaT (immortalized human keratinocyte) was acquired from the Rio de Janeiro cell bank (BCRJ, Rio de Janeiro, Brazil). The cells were grown in 25 cm^2^ culture bottles in an incubator at 37°C with 5% CO_2_, using Dulbecco’s Modified Eagle Medium (DMEM) high glucose as a nutritional source and RPMI for HaCaT and MDA-MB-231 cell lines respectively. Both mediums were supplemented with 10% fetal bovine serum (Gibco^®^) plus penicillin at 100 UI/mL and streptomycin at 100 μg/ml (Gibco^®^) (de Campos et al., 2010; Melo et al., 2018).

### 2.4 Cell viability assay

Cell viability was assessed by 3-(4,5-dimethylthiazol-2-yl)-2,5-diphenyltetrazolium bromide (MTT) methodology. Briefly, 100 μL of both MDA-MB-231 and HaCaT cell lines containing 1.5 × 10^4^ and 1 × 10^4^ cells were pre-cultured in 96-well plates for 24 and 48 h, respectively. After 80% confluence, different concentrations of mother tinctures varying from 0.5 to 3.0% v/v were added in each well (Mosmann, 1983). The percentage of viable cells was calculated in relation to control cells (untreated and treated with ethanol solvent) using mean values from at least three independent experiments performed in triplicate. The IC_50_ value was calculated using GraphPad Prism^®^ 9.0.

### 2.5 Glucose consumption and lactate production

MDA-MB-231 glucose consumption was performed assessing the glucose content that remained in the culture media using an enzyme system containing glucose oxidase (Accu-Check Active^®^ - Roche). For this, four experimental groups were performed, regarding each MT, as following: *V. album* MT from *Abies alba* at 1 and 2% v/v; *V. album* MT from *Quercus petraea* at 0.5 and 1% v/v, for 48 and 72 h of incubation, at cellular cultivation conditions. Lactate production was evaluated as described previously (Zancan et al., 2010) assessing the lactate released in the culture media. The generation of NADH was measured at 340 nm in the presence of 1 U/ml lactate dehydrogenase (Sigma-Aldrich, St-Louis, MO, United States) by incubating the media in the presence of the enzyme and NAD^+^. The glucose uptake and the lactate production were calculated in relation to ethanol solvent at 1 and 2% (MT *Abies alba*); and at 0.5 and 1% (MT *Quercus petrea*), in order to discount the solvent effect in cellular glucose consumption. Statistical analysis and non-linear regression were performed using the software SigmaPlot 10.0 integrated with SigmaStat 3.1 packages (Systat, CA, United States) and GraphPad Prism^®^ 9.0.

### 2.6 Spectrophotometric assay for enzyme activity

MDA-MB-231 cells were seeded in 12 well plates at 12 × 10^4^ and 6 × 10^4^ cells/well for 48 and 72 h of the treatment respectively. After cellular confluence, MTs or the vehicle control (alcohol at the same concentration of the MT) were added, and plates incubated for 48 or 72 h. Controls without any treatment were done. Then, medium was removed, and cells were detached from the plates by trypsinization. Protein concentrations of cell lysates were measured, and the glycolytic enzyme activities were evaluated. Hexokinase (HK), pyruvate kinase (PK), phosphofructokinase (PFK-1) and glucose-6-phosphate dehydrogenase (G6PDH) activities were assayed as previously described (Zancan et al., 2010). Briefly, for HK activity, a coupled enzyme system containing 0.2 U/ml glucose-6-phosphate dehydrogenase (Sigma-Aldrich, St-Louis, MO, United States) was used to oxidize the glucose-6-phosphate formed by HK reaction and reduce NAD^+^. For PFK-1 activity, the coupled enzyme system contained 1 U/ml aldolase (Sigma-Aldrich, St-Louis, MO, United States), 2 U/ml triose-phosphate isomerase (Sigma-Aldrich, St-Louis, MO, United States) and 1 U/ml α-glycerophosphate dehydrogenase (Sigma-Aldrich, St-Louis, MO, United States) to reduce NAD^+^ coupled to PFK-1 was used. For PK activity, the same rational was applied and the coupled enzyme contained 1 U/ml lactate dehydrogenase (Sigma-Aldrich, St-Louis, MO, United States) to oxidize NADH coupled to PK activity was added. For G6PDH activity, NAD^+^ reduction coupled to glucose-6-phosphate oxidation was directly measured. NADH oxidation or NAD^+^ reduction was followed by measuring the absorbance at 340 nm in a microplate reader (VICTOR 3, PerkinElmer). Reactions were initiated by the addition of an aliquot of cellular homogenate. Blanks with none of the coupled enzymes were also performed as controls to exclude non-specific oxidation/reduction reactions (Zancan et al., 2010). Each curve was performed in triplicate. Statistical analysis and non-linear regression were performed using the software SigmaPlot 10.0 integrated with SigmaStat 3.1 packages (Systat, CA, United States) and GraphPad Prism^®^ 9.0.

### 2.7 Quantification of viscotoxins in *Viscum album* mother tinctures by high performance liquid chromatography

Each MT was purified by solid-phase extraction (Bakerbond, carboxylic acid, wide pore SPE column). Subsequently, the samples were eluted with 5 ml of 0.4 M acetic acid, and the acid eluates obtained were injected into a high-performance liquid chromatography with diode array detection (HPLC-DAD) to quantify the viscotoxins. The column used was a Nucleosil C_18_ AB, 125 × 4 mm × 5 µm with a guard column. Mobile phase A was water with 0.1% trifluoroacetic acid (TFA), mobile phase B was 60/40 acetonitrile/water with 0.1% TFA. Gradient elution was as follow: 38% B to 42% B in 9 min, 50% B in 2 min, 50% to 54% B in 8 min; flow rate: 1 ml/min; injection volume: 200 μL; detection: UV at 210 nm. The total viscotoxin content in (µg/ml) and mg/g of fresh material (FM) were calculated as the sum of each viscotoxin isoform, considering the following elution order: B, U-PS, A1, 1-PS, A2, A3 (Schaller et al., 1998; Holandino et al., 2020). The quantitative calculation was done with all five mother tinctures of MT M, MT Q and MT U. For MT A and MT P it was performed only with three samples because it was not possible to harvest from the same host tree in both seasons. The analysis of variance (two-way ANOVA) followed by Bonferroni post hoc test was performed using the GraphPad Prism^®^ 9.0 software. Differences were considered significant when *p* < 0.05.

### 2.8 *Viscum album* mother tinctures analysis by liquid chromatography coupled to high-resolution mass spectrometry

Initially, 1.0 g of mother tincture was weighed and transferred to a 5 ml volumetric flask where a solution 9:1 acetonitrile/water with 0.1% v/v formic acid was added. After homogenization, the solution was centrifuged at 7,056 g for 15 min and then 200 µl of each supernatant were removed for analysis in a DionexUltiMate 3000 liquid Chromatography coupled to a hybrid Quadrupole-Orbitrap high-resolution mass spectrometer (Thermo Q-Exactive Plus) equipped with an electrospray ionization source (ESI). Pooled quality control samples (QC) containing 10 µl of each MT were also prepared. Chromatography separation was performed in a reversed-phase column (Hypersil Gold C_18_, 100 mm × 2.1 mm × 3.0 μm; Thermo Fisher Scientific). Water with 0.1% v/v of formic acid (A) and acetonitrile with 0.1% v/v of formic acid (B) were used as the mobile phases in an elution gradient of: 1) 0–1 min, 10% B; 2) 1–16 min, 10–95% B; 3) 16–18 min, 95% B; 4) 18–18.1 min, 95–10% B; 5) 18.1–22 min 10% B. The flow rate was 0.350 μl/min and the injection volume 5 µl. Mass spectra were acquired simultaneously in negative and positive ionization modes in the *m/z* range of 100–1000 at a resolution of 35 k (FWHM) followed by sequential mass spectrometry (MS/MS) experiments ddMS2 top 3 (where the three most intense ions of each scan were fragmented automatically). The mass spectrometry conditions were the following: spray voltage 3.9 kV for ESI (+) or 3.6 kV for ESI (-), ion transfer capillary 300°C, sheath, and auxiliary gases 45 and 15 arbitrary units, respectively. Data were analyzed by the Xcalibur 2.0.7 program (Thermo Scientific, Bremen, Germany) (Holandino et al., 2020).

### 2.9 A pre-processing of the LC-HRMS data and multivariate data analysis

A pre-processing of the data was performed to remove ions from the electronic noise of the equipment, ions from the mobile phase and/or that did not reach certain parameters of intensity, adequate format, and isotopic profile. The raw data files were submitted to peak detection, deconvolution, normalization, and alignment processes using the freely available MZmine 2.35 software according to the parameters shown in Supplementary Table S1 (Pluskal et al., 2010; Olivon et al., 2017). The obtained peak lists were generated according to the following observations: samples in columns and variables (i.e., markers of given retention time and *m/z*) in rows were exported to Metaboanalyst 5.0 web server (https://www.metaboanalyst.ca/) for the principal component analysis (PCA) and the partial least squares discriminant analysis (PLS-DA) (Chong et al., 2019). Parameters for multivariate analysis in MetaboAnalyst 5.0 can be found in the Supplementary material (Supplementary Table S3 and Supplementary Figures S1, S2). Data were auto scaled prior to multivariate analysis. PLS-DAs models were validated using leave-one-subject-out cross-validation with R2 and Q2 metrics.

### 2.10 Metabolite annotation

Raw data files were converted to ABF format using the ABF converter software for use in the MSDial (https://www.reifycs.com/AbfConverter/accessed on 18 June 2020). Compound annotation was performed by MSDial 4.70 comparing the aligned *m/z* ions and their deconvoluted MS/MS spectra to those uploaded to the MassBank of North America (http://mona.fiehnlab.ucdavis.edu/accessed on 25 September 2021) and the NIST 2014 MS/MS library (Tsugawa et al., 2015). In some cases, the structure suggestion was based on the fragmentation profile and the neutral loss. The parameters used are identified in Supplementary Table S2. The use of metabolomic approach has been recently used to show the impact of host-tree and season variation in *Viscum album* metabolite’s profiles (Jäger et al., 2021; Segneanu et al., 2022).

## 3 Results

### 3.1 Cell cytotoxicity and glycolytic enzymatic activity triggered by *Viscum album* mother tinctures

**FIGURE 1:**
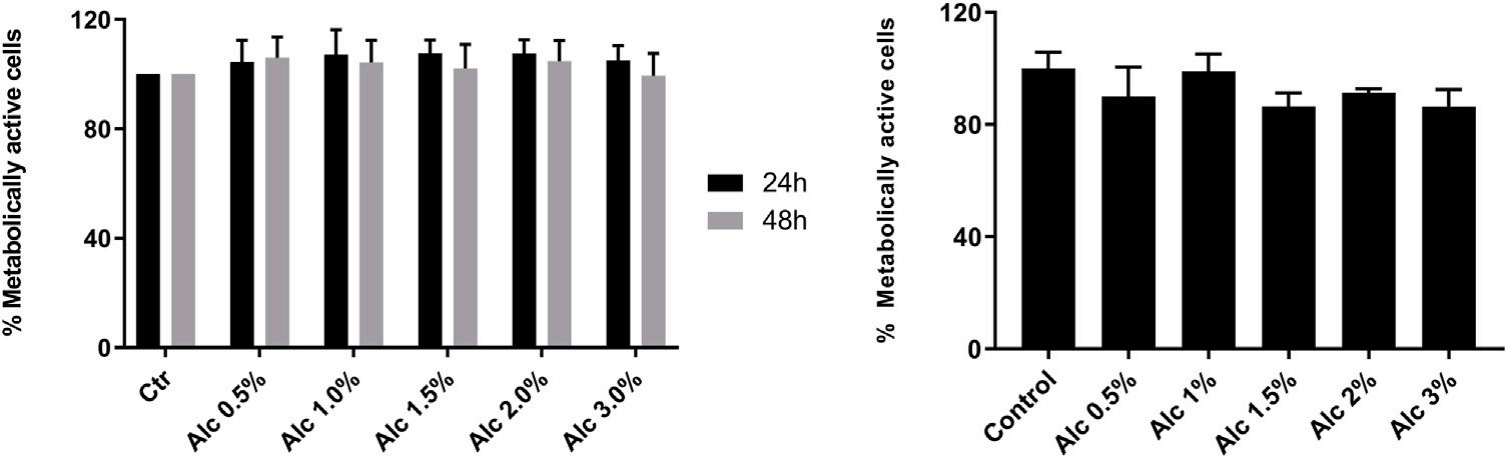
Cytotoxic effects induced by ethanol solvent on MDA-MB-231 and HaCaT cell lines. MTT assay was done after two times of incubation, 24 h (black bars) and 48 h (grey bars), as following: 24 and 48 h s for MDA-MB-231 cells (left graph); 24 h s for HaCaT cells (right graph). The ethanol range concentrations varied from 0.5 to 3.0% v/v (Alc). Control groups: untreated cells incubated in culture medium for 24 and 48 h s (MDA-MB-231) and for 24 h s (HaCaT). The results are expressed as the mean ± standard error from three independent experiments, done in triplicate, in relation to control groups (untreated and treated with ethanol).

**FIGURE 2:**
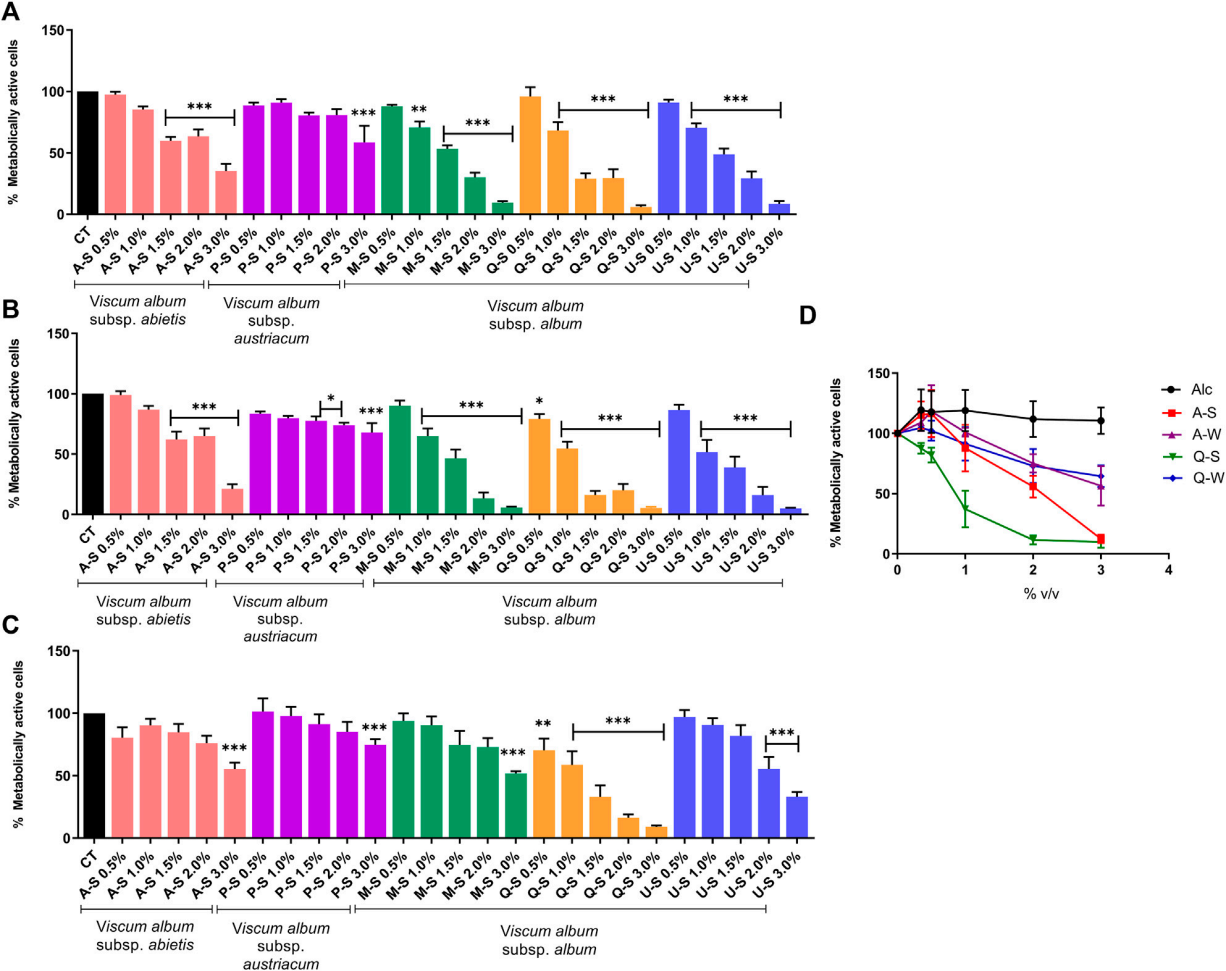
Dose-response effect of *V. album* mother tinctures on MDA-MB-231 and HaCaT cell lines proliferation assessed by MTT. **(A)** MDA-MB-231 summer-dose effects after 24 h of treatment with MTs. **(B)** MDA-MB-231 summer-dose response curve after 48 h of treatment with MTs **(C)** HaCaT summer-dose response effects after 24 h of treatment with MTs. **(D)** MDA-MB-231 dose response curves after 72 h of treatment with summer and winter MTs (A-S/W; Q-S/W). The final concentrations varied from 0.5 to 3.0% v/v. The results are calculated as the mean ± standard error from three independent experiments, done in triplicate, in relation to control groups (untreated and treated with ethanol). *p < 0.05; **p < 0.001; ***p < 0.0001 obtained with one-way ANOVA/Dunnet’s post hoc test. Legend symbols: (CT) control untreated cells; (Alc) cells incubated with ethanol solvent; *V. album* subsp. *album* growing on *Malus domestica* (M-S/W), *Quercus petraea* (Q-S/W) and *Ulmus carpinifolia* (U-S/W); *V. album* subsp. *abietis* growing on *Abies alba* (A-S/W); and *V. album* subsp. *austriacum* growing on *Pinus sylvestris* (P-S/W). Samples were harvested in summer (S) and winter (W) seasons.

**FIGURE 3:**
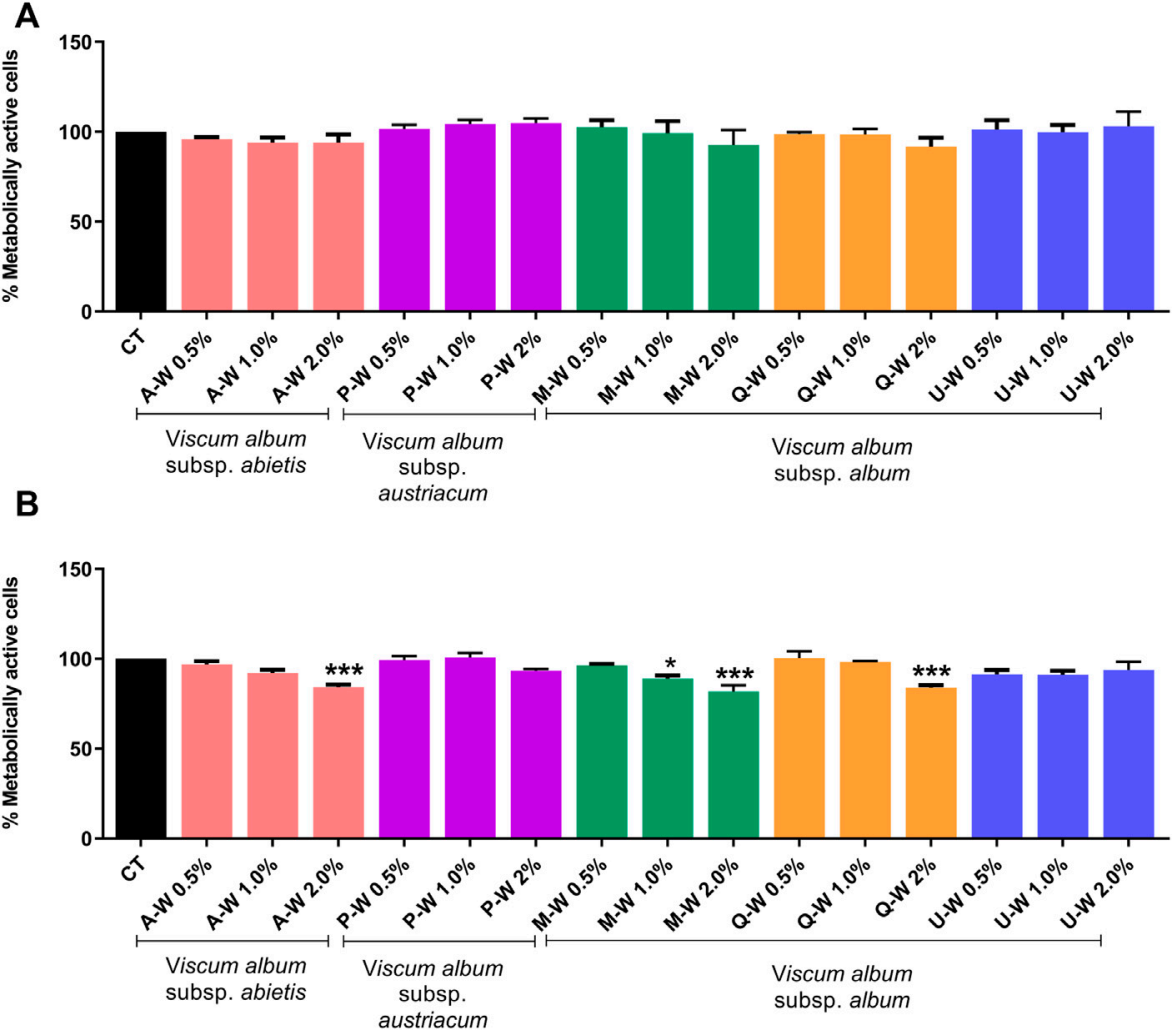
Dose-response effect of *V. album* mother tinctures on the proliferation of MDA-MB-31 cell line assessed by MTT. **(A)** MDA-MB-231 winter-dose response effects after 24 h of treatment with MTs. **(B)** MDA-MB-231 winter-dose response effects after 48 h of treatment with MTs. The final MTs concentrations tested were: 0.5; 1.0; 2.0% v/v. The results are expressed as the mean ± standard error from three independent experiments done in triplicate, in relation to control cells (untreated and treated with ethanol). *p < 0.05; ***p < 0.0001 obtained with one-way ANOVA/Dunnet’s post hoc test. Legend symbols: (CT) control untreated cells; *V. album* subsp. *album* growing on *Malus domestica* (M–W), *Quercus petraea* (Q–W) and *Ulmus carpinifolia* (U–W); *V. album* subsp. *abietis* growing on *Abies alba* (A–W); and *V. album* subsp. *austriacum* growing on *Pinus sylvestris* (P–W). Samples were harvested in winter (W) season.

To investigate the cytotoxic effects of *V. album* mother tinctures, MDA-MB-231 and HaCaT cell lines were treated with increasing concentrations of all MT, and the mitochondrial metabolism was evaluated by MTT assay. Initially, the ethanol toxicity was evaluated and, as described before by Holandino et al. (2020), no cytotoxic effects were detected by ethanol solvent in both cells (Figure 1). However, summer and winter MT samples triggered different responses (Figure 2). The summer MT at 24 and 48 h had greater activity than winter MT (Figures 2A,B, 3 A,B). The cytotoxicity evaluated in MDA-MB-231 (Figures 2A,B) and in HaCaT (Figure 2C) cell lines was dose and time-dependent for A-S, M-S, Q-S, and U-S. *V. album* subsp. *album* was the most cytotoxic MT for MDA-MB-231, with IC_50_ < 2% v/v at 24 and 48 h (Figures 2A,B). However, among the three host trees of the *V. album* subsp. *album* there was a difference in the cellular viability. According to Figure 2, the MT produced with *V. album* subsp. *album* harvested from *Quercus petraea* was the most cytotoxic, with IC_50_ of 1.48 and 1.23% v/v at 24 and 48 h, respectively. The cytotoxic analysis indicated that mistletoe collected from conifers (*Viscum album* subsp*. abietis* and *Viscum album* subsp*. austriacum*) presented different profiles. MT A-S had an IC_50_ of 2.38 and 2.18% v/v at 24 and 48 h, respectively. However, the lowest cytotoxic activity was related to MT P-S, with statistically significance differences detected in the following concentrations tested: 3% v/v (*p* < 0.0001) after 24 h and 48 h; 1.5–2% (*p* < 0.05) after 48 h of incubation (Figures 2A,B).

Since the cytotoxic effects were directly influenced by season (Figures 2, 3), the selectivity index (SI) was calculated only with summer MT, in relation to the normal HaCaT cell line, at 24 h of incubation. The cytotoxic effects on normal cells were compared with those induced by Q-S and A-S in breast cancer cells (Figures 2A,C). The SI was 1.0 and 1.7 for Q-S and A-S, respectively, highlighting that *V. album* from *Quercus petraea* was not selective and had a similar cytotoxic effect for both normal and tumor cell lines. On the other hand, the A-S was more cytotoxic for the tumor cell line than for the normal one.

**FIGURE 4:**
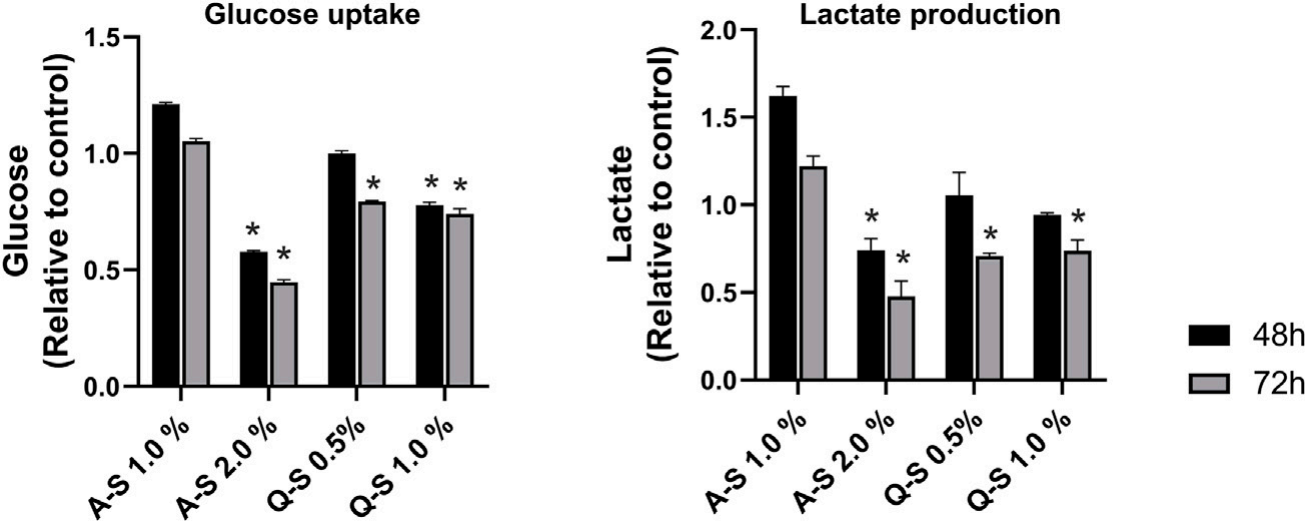
Glucose uptake (left graph) and lactate production (right graph) by MDA-MB-231 cells treated with *Viscum album* summer mother tinctures for 48 h (black bars) and 72 h (grey bars) of the treatment with A-S (1.0% and 2.0% v/v) and Q-S (0.5% and 1% v/v). Plotted values are relative to the control (ethanol vehicle) at the same concentration of the summer mother tinctures, *p < 0.05 calculated by nonparametric Mann-Whitney test. Legend symbols: *V. album* subsp. *abietis* growing on *Abies alba* in summer (A–S); *V. album* subsp. *album* growing on *Quercus petraea* in summer (Q–S).

The effects of Q-S and A-S MT on glucose taken up by the MDA-MB-231 cell line were evaluated, and the results are shown in Figure 4-left. Mother tincture A-S at 2.0% v/v reduced approximately 50% of the glucose uptake after 48 h of treatment, with more significant effects after 72 h of sample incubation. The MT Q-S at 1.0% v/v inhibited approximately 20% of the glucose uptake in both incubation times analyzed. A similar result was observed on Q-S 0.5% after 72 h. All these MT effects on glucose uptake, and under its metabolism, promoted a reduction in lactate cellular production. Because of this, a significant reduction (*p* < 0.05) in extracellular lactate levels was observed, especially after 72 h of incubation with Q-S 0.5%, Q-S 1.0%, and A-S 2.0% v/v (Figure 4-right graph).

**FIGURE 5:**
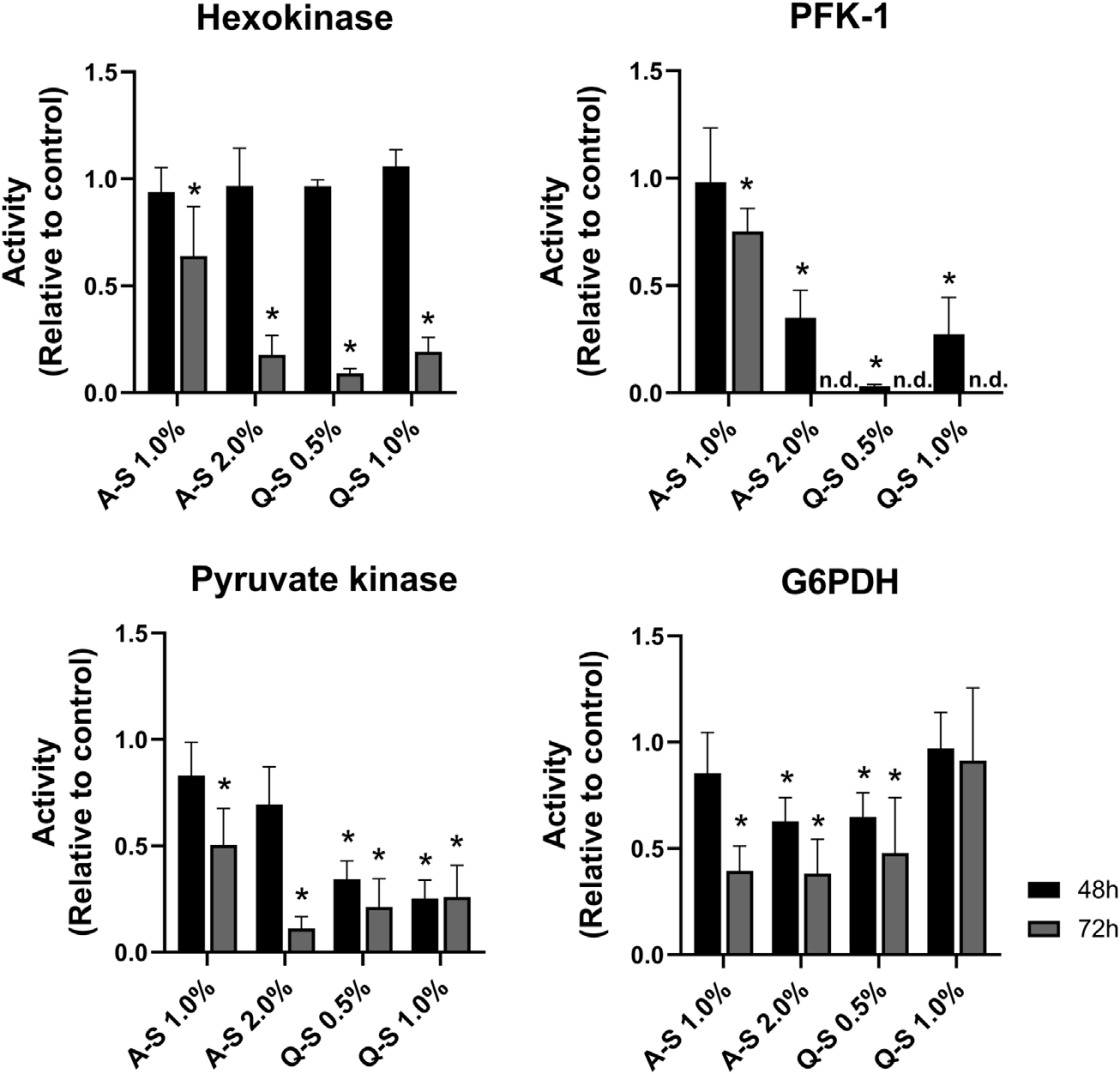
Effect of *V. album* summer mother tinctures on the MDA-MB-231 glycolysis and pentose phosphate pathway enzymes. HK, PFK-1, PK (glycolytic enzymes), and G6PDH (Pentose Phosphate Pathway) were analyzed after 48 h (black bars) and 72 h (grey bars) of the treatment with A-S (1.0% and 2.0% v/v) and Q-S (0.5% and 1% v/v). Mean values ±standard error of two independent experiments (*n* = 2). *p ≤ 0.05 were compared to control (ethanol at the same concentration of MTs) by nonparametric Mann-Whitney test. Legend symbols: *V. album* subsp. *abietis* growing on *Abies alba* in summer (A–S); *V. album* subsp. *album* growing on *Quercus petraea* in summer (Q–S).

Following the alterations in the glucose uptake and lactate production, the activities of glycolytic enzymes were evaluated (Figure 5). The results indicated MT-promoted inhibition of glucose metabolism in MDA-MB-231 breast cancer cells. Regarding the first step of the glycolytic pathway, HK activity was significantly inhibited by MT (A-S 2.0%, Q-S 0.5%, and Q-S 1.0% v/v) after 72 h of incubation. Additionally, PFK-1 activity was dramatically impaired at 48 h, and at 72 h it was not possible to detect enzymatic activity. This suggests that this step enabled strong inhibition of the glycolytic pathway (Figure 5). Subsequently, the effects of MT (A-S and Q-S) on PK activity were analyzed. After 72 h, A-S incubation inhibited the enzymatic activity in a dose-dependent manner. Nevertheless, MT Q-S promoted a homogeneous and constant inhibition of this kinase for both concentrations and treatment times.

The effects of MT on the Pentose Phosphate Pathway (PPP) were analyzed by evaluating the activity of Glucose-6-phosphate dehydrogenase (G6PDH). Figure 5 shows a great response variation, which does not suggest direct effects on this enzyme activity.

Since biological activities of *V. album* mother tinctures are related to the host trees and the influence of the seasons, the metabolome profile and, consequently chemical variability of several summer and winter preparations were evaluated by liquid chromatography.

### 3.2 Chemical variability of the *Viscum album* subsp. mother tinctures

#### 3.2.1 Mother tinctures and viscotoxin content

**FIGURE 6:**
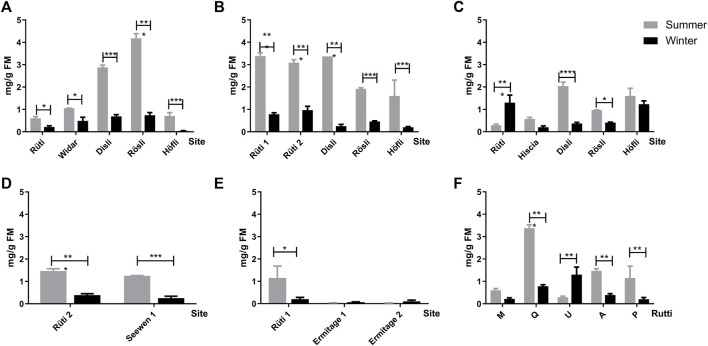
Amount of viscotoxin in mg/g of fresh material (FM) of *V. album* mother tinctures prepared with plants harvested in summer (grey bars) and in winter (black bars), in the following locations: Rütti, Widar, Disli, Rösli, Höfli, Hiscia, Seewen, Ermitage. MTs were prepared with mistletoe collected from the following host trees: *Malus domestica*. **(A)** *Quercus* sp. **(B)** *Ulmus carpinifolia*. **(C)** *Abies alba*. **(D)** and *Pinus sylvestris* **(E)**. Comparison of the viscotoxin content from the different host trees in the same location, Rüti. **(F)** The numbers added to Rütti and Ermitage locations represent *V. album* from different host-trees harvested in the same location. *p ≤ 0.05; ***p < 0.0001.

The viscotoxin (VT) contents were evaluated by HPLC-DAD according to the *V. album* subspecies, host tree, and season (Figure 6). In general, subspecies of *V. album* harvested in summer had the highest amount of these proteins. Regarding deciduous trees, they were able to produce the highest concentration of viscotoxin in mg/g fresh material (FM), and this profile was mostly related to Disli and Rösli (*Malus domestica*), and Rütti (*Quercus* sp.) harvesting sites (Figures 6A–C). Additionally, *V. album* MT (*Malus domestica*) from Rösli produced the maximum VT amount detected, which was around 4 mg of VT/g FM (Figure 6A). On the other hand, VT contents of MT from conifers were smaller than deciduous host trees (Figures 6D,E). The analyses of VT from host trees at the same location (Rütti) showed summer season positive influence on this protein production, except for *V. album* MT from *Ulmus carpinifolia* (Figure 6F).

#### 3.2.2 Untargeted metabolome analysis focusing on host tree influence

**FIGURE 7:**
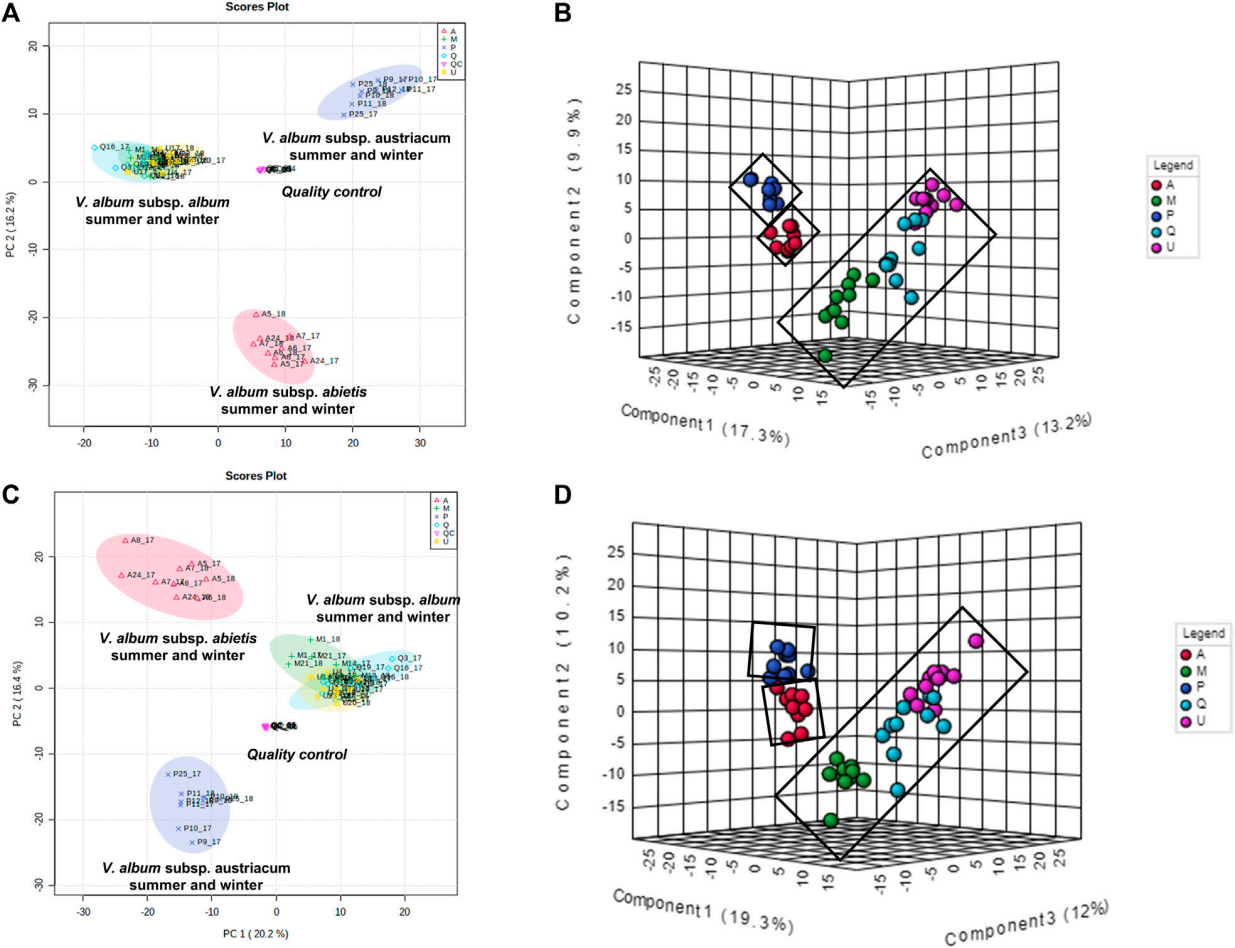
Multivariate analysis from LC-HRMS data of *V. album* mother tinctures. Discrimination of the samples in relation to *V. album* host trees. **(A)** PCA 2D score plot of the negative ESI mode. **(B)** PLS-DA 3D score plot of the negative ESI mode **(C)** PCA 2D score plot of the positive ESI mode. **(D)** PLS-DA 3D score plot of the positive ESI mode.

To better understand how the metabolic profiles of small molecules from mother tinctures differed, unsupervised and supervised multivariate analyses were conducted. After the pre-processing and data pre-treatment steps, the final dataset used for the multivariate analysis consisted of 56 samples × 850 features and 56 samples × 685 features (*m/z* and retention time) for the negative and positive ESI modes, respectively. Firstly, PCA plots were able to demonstrate the natural clustering of samples into three groups related to the botanical subspecies for both negative and positive electrospray ionization modes (Figures 7A,C). The pooled QC samples were tightly clustered, showing a good reproducibility of the LC-HRMS system (Figures 7A,C). In the PCA, the first principal component (PC1) explained 20.2% of the total co-variance for both ionization modes, whereas the second principal component (PC2) accounted for 16.2 and 16.4% (for negative and positive modes, respectively) of the total variability of the data set.

**FIGURE 8:**
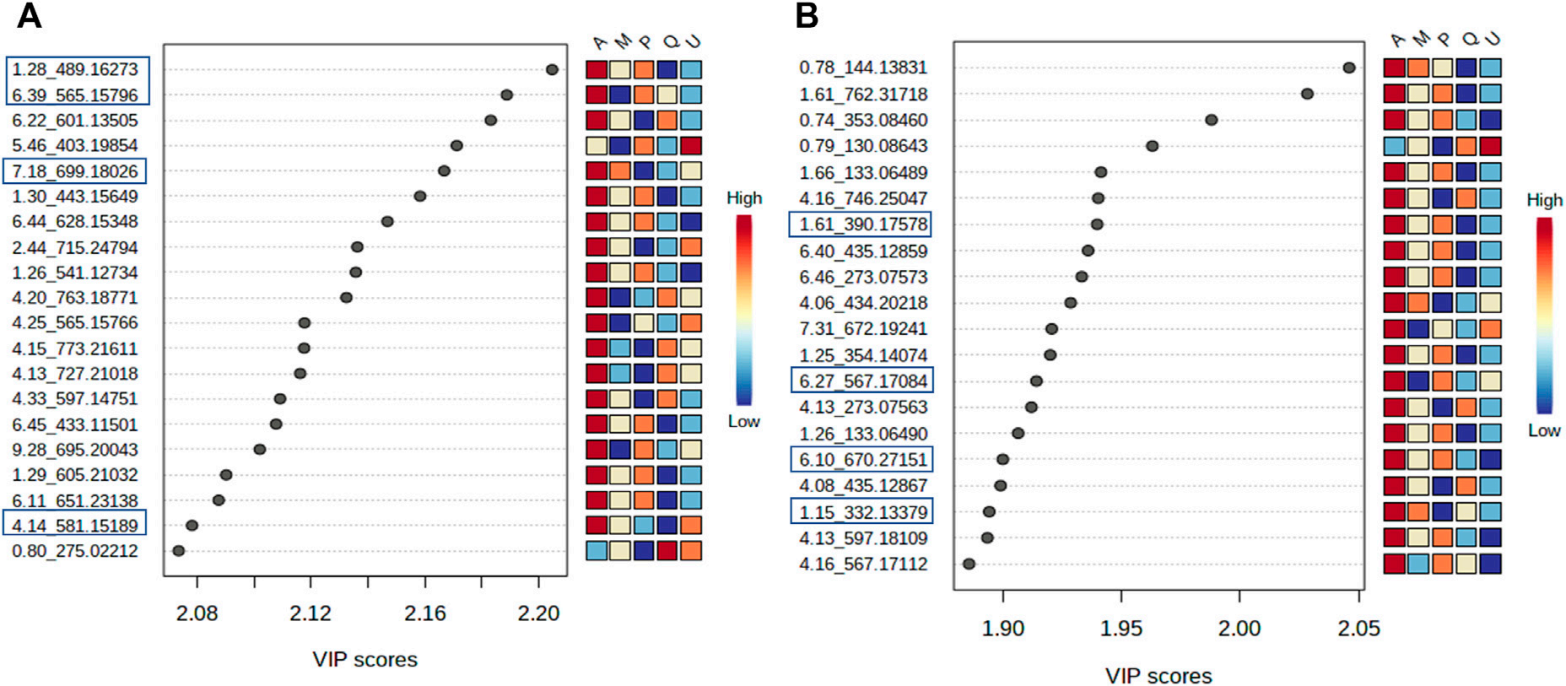
Variables important projection (VIPs) from PLS-DA of *V. album* mother tinctures. Discrimination of the samples in relation to *V. album* host trees. **(A)** VIP of the negative ESI mode and. **(B)** VIP of the positive ESI mode. Blue rectangles: annotated VIP.

Then, PLS-DA was conducted considering the host tree groups. The 3D score plot showed a clear subspecies separation in three clusters (Figures 7B,D). However, it was possible to identify five different groups related to each host tree evaluated in this study. Then, the main compounds responsible for host tree discrimination were determined by variable important projection (VIP) in the negative (Figure 8A) and positive ionization modes (Figure 8B). The R2/Q2 metrics for PLS-DA were >0.8 for both modeling processes, increasing from component 2 to 5, which demonstrated that the data was a good fit (Worley and Powers, 2012).

VIP scores were generated, and the most important variables for the MT separation according to their host tree were identified. The annotation was based on the comparison of fragmentation data (Supplementary Figures S3,S4) obtained by LC-HRMS analysis, with MS/MS of the mass banks described in materials and methods, considering the error <8 ppm calculated in relation to the exact mass.

In the negative ionization mode (Figure 8A), it was observed that there were increased levels of four molecules in the A group (*V. album* subsp. *abietis*) comprising three flavonoids and one terpene, respectively: flavanone-hexose-pentose; naringenin-pentose-hexose; 2-hydroxy-4-(6-hydroxy-5,7-dimethoxy-4-oxo-4H-chromen-3-yl) phenyl6-Ohexopyranosylhexopy-ranoside; 6′-O-β-D-apiofuranosylsweroside) (Table 1).

**TABLE 1:** Annotated VIP responsible for *Viscum album* host tree discrimination in negative and positive ionization modes.

Discrimination in the negative ESI mode						
Rt	Molecular formula	Adduct	Theoretical *m/z*	Experimental *m/z*	Error (ppm)	Annotated compound (chemical class)
1.28	C_21_H_30_O_13_	[M-H]^−^	489.16026	489.16273	-5.05	6′-O-β-D-Apiofuranosylsweroside (Monoterpene)
4.14	-	-	-	581.15189	-	Flavanone base-hexose-pentose
6.39	C_26_H_30_O_14_	[M-H]^−^	565.15628	565.15796	-2.97	Naringenin-pentose-hexose (Flavanone)
7.18	C_29_H_34_O_17_	[M + CHO_2_]^−^	699.17836	699.18026	-2.71	2-Hydroxy-4-(6-hydroxy-5,7-dimethoxy-4-oxo-4H-chromen-3-yl)phenyl 6-O-hexopyranosylhexopyranoside (Isoflavone)
**Discrimination in the positive ESI mode**						
1.15	C_14_H_18_O_8_	[M + NH_4_]^+^	332.13399	332.13379	0.60	(4-(beta-D-Glucopyranosyloxy)phenyl)acetic acid (Organic acid)
1.61	C_27_H_24_O_9_	[M + NH_4_]^+^	390.17530	390.17578	-1.23	Syringin = eleutheroside B (phenylpropanoid glycoside)
6.10	C_31_H_40_O_15_	[M + NH_4_]^+^	670.27219	670.27151	1.02	[5-hydroxy-6-[2-(4-hydroxy-3-methoxyphenyl)ethoxy]-2-(hydroxymethyl)-4-(3,4,5-trihydroxy-6-methyloxan-2-yl)oxyoxan-3-yl] (E)-3-(4-hydroxy-3-methoxyphenyl)prop-2-enoate (Phenylpropanoid)
6.27	C_26_H_30_O_14_	[M + H]^+^	567.17083	567.17084	-0.02	Naringenin-pentose-hexose (Flavanone)

In parallel, the positive ionization mode showed four compounds that are important to group A discrimination, namely, one organic acid, two phenylpropanoids and one flavanone, respectively: (4-(β-D-glucopyranosyloxy) phenyl) acetic acid; Syringin [5-hydroxy-6-[2-(4-hydroxy-3-methoxyphenyl) ethoxy]-2-(hydroxymethyl)-4-(3,4,5-trihydroxy-6-methyloxan-2-yl) oxyoxan-3 -yl] (E)-3-(4-hydroxy-3-methoxyphenyl) prop-2-enoate; naringenin-pentose-hexose (Table 1).

As shown in the VIP score plot (Figures 8A,B), naringenin-pentose-hexose was a flavanone annotated with *m/z* 565 in negative, and *m/z* 567 in positive ionizations modes, which demonstrates its importance for the group A discrimination.

#### 3.2.3 Untargeted metabolome analysis focusing on subspecies influence

**FIGURE 9:**
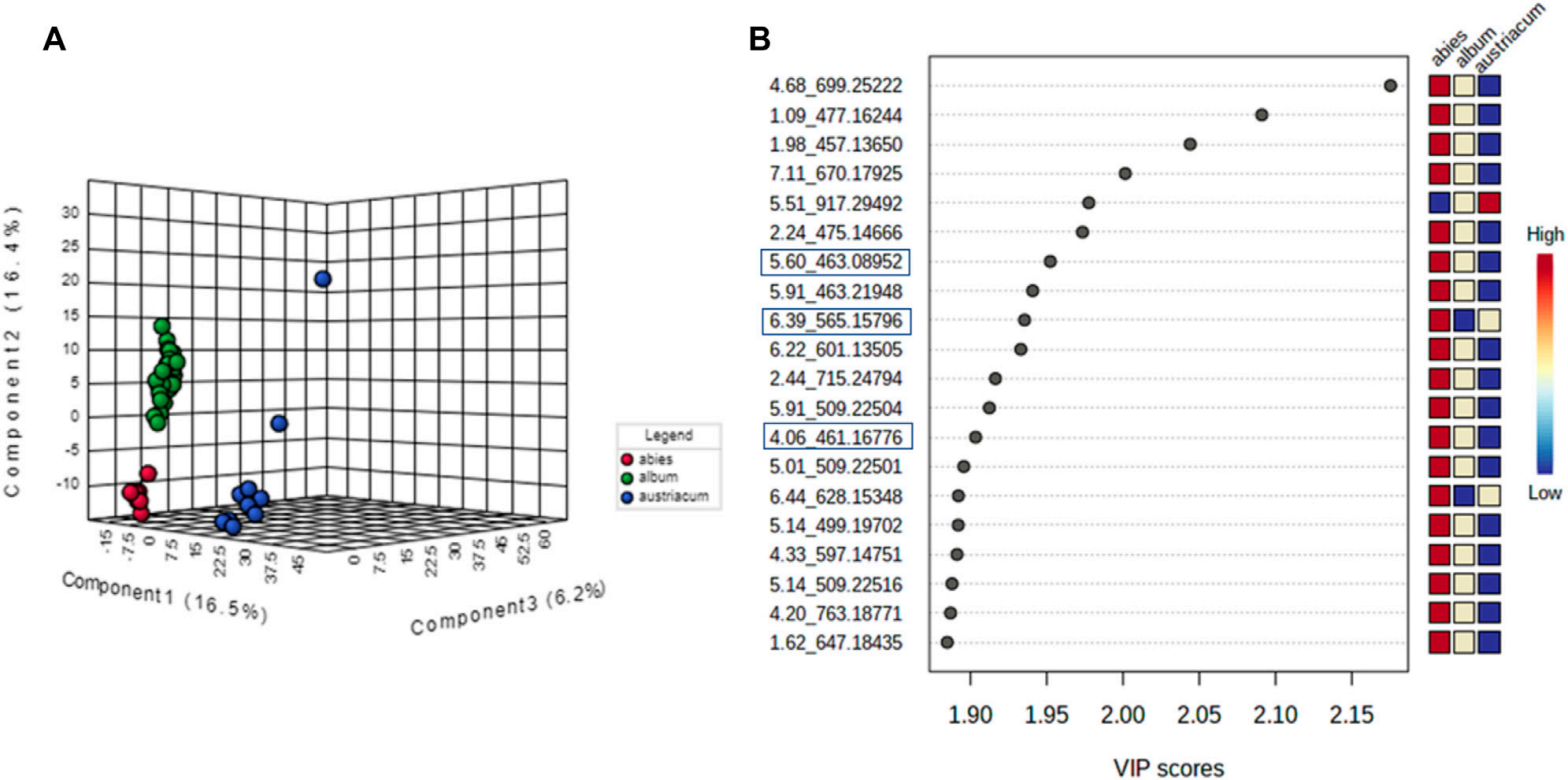
PLS-DA of *V. album* mother tinctures. Discrimination of the samples in relation to *V. album* subspecies. **(A)** PLS-DA 3D score plot of the negative ESI mode and. **(B)** VIP of the negative ESI mode. Blue rectangles: annotated VIP.

Regarding the biological results obtained, and the need to understand the MT cytotoxic differences, a new supervised analysis considering exclusively the subspecies groups was performed. The first three components of PLS-DA explained 49% of the total co-variance for the negative ionization model (Figure 9A). The VIP score plot indicated seventeen compounds related to *V. album* subsp. *abietis* and *V. album* subsp. *album* with higher intensity when compared to *V. album* subsp. *austriacum* (Figure 9B). Additionally, based on VIPs, three of these compounds were annotated (SupplementaryFigure S5) in the negative ionization mode, as follows: 2-Phenylethyl 6-O-pentopyranosylhexopyranoside, hyperoside, and naringenin-pentose-hexose (Table 2).

**TABLE 2:** Annotated VIP responsible for *Viscum album* subspecies discrimination in negative ionization mode.

Rt	Molecular formula	Adduct	Theoretical *m/z*	Experimental *m/z*	Error (ppm)	Annotated compound (chemical class)
4.06	C_19_H_28_O_10_	[M ^+^ CHO_2_]^−^	461.16535	461.16776	-5.22	2-Phenylethyl 6-O-pentopyranosylhexopyranoside (Sugar derivative)
5.60	C_21_H_20_O_12_	[M-H]^−^	463.08819	463.08952	-2.87	Hyperoside (Flavonol)
6.39	C_26_H_30_O_14_	[M-H]^−^	565.15628	565.15796	-2.97	Naringenin-pentose-hexose (Flavanone)

#### 3.2.4 Untargeted metabolome analysis focusing on season influence

**FIGURE 10:**
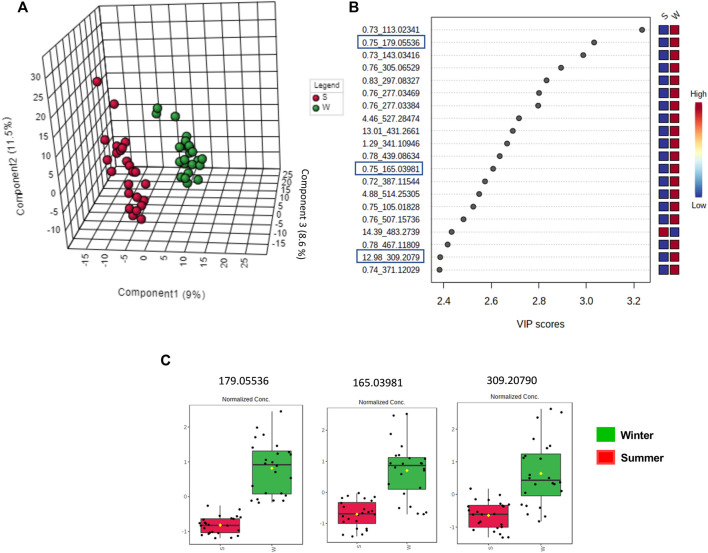
Multivariate analysis from LC-HRMS data of *V. album* mother tinctures in negative ESI mode. Discrimination of the samples in relation to *V. album* season harvesting. **(A)** PLS-DA 3D score plot. **(B)** VIP scores. **(C)** Box plot graphs of the annotated metabolites (VIP) responsible for discriminating winter (green) and summer (red) samples. Blue rectangles: annotated VIP.

**FIGURE 11:**
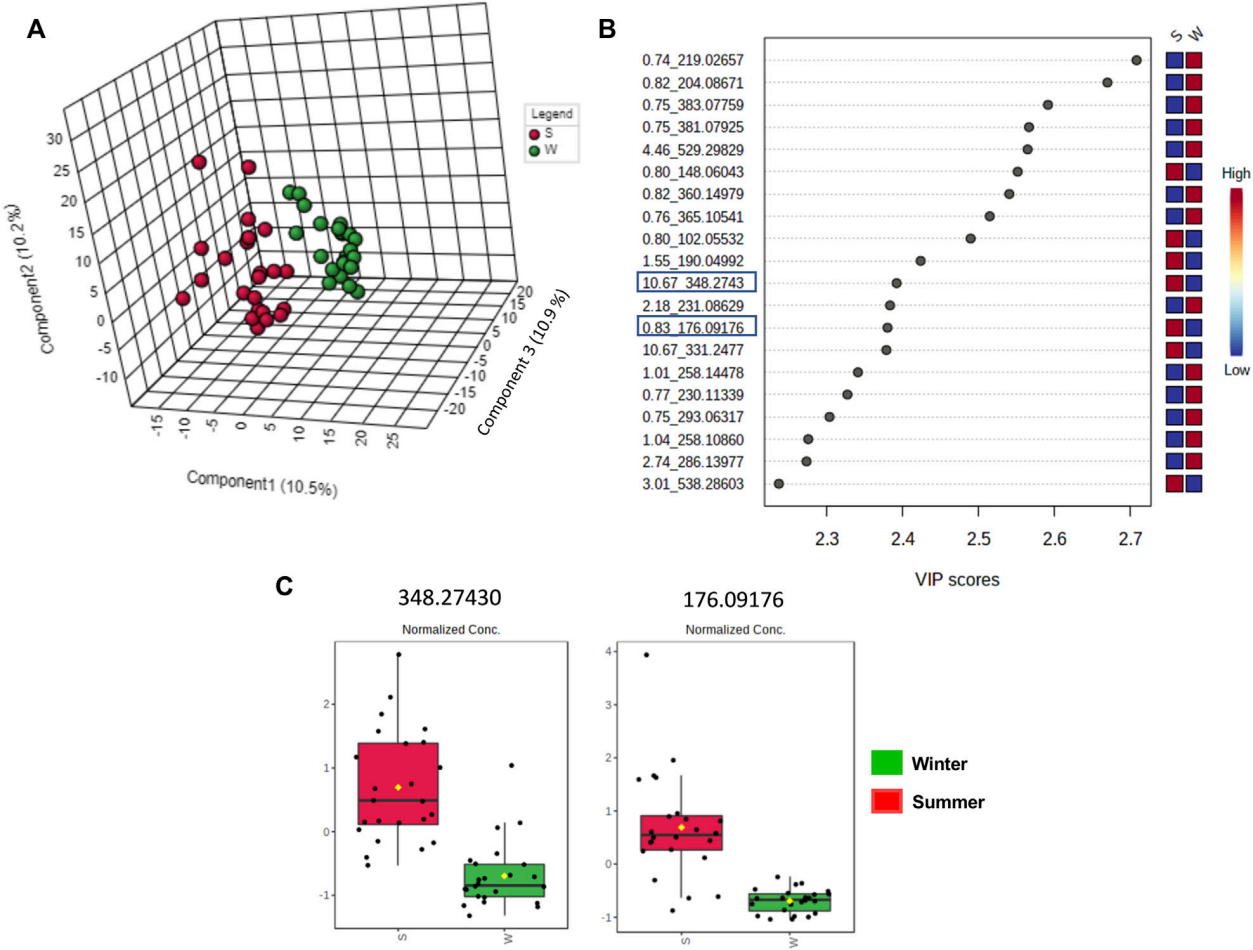
Multivariate analysis from LC-HRMS data of *V. album* mother tinctures in positive ESI mode. Discrimination of the samples in relation to *V. album* season harvesting. **(A)** PLS-DA 3D score plot. **(B)** VIP end point **(C)** Box plot graphs of the annotated metabolites (VIP) responsible for discriminating winter (green) and summer (red) samples. Blue rectangles: annotated VIP.

The clustered pattern addressed by previous PLS-DA score plots (Figures 7–9) was not able to differentiate the *V. album* mother tinctures metabolome in relation to the harvested seasons. Hence, a new supervised analysis was conducted focusing on the plant material harvesting season. As a result, the first three principal components analysis explained approximately 30% of the data co-variance in both ionization modes (Figures 10A, 11A). It was possible to identify those variables responsible for the summer/winter samples discrimination in the negative (Figures 10B,C) and positive (Figures 11B,C) ionization modes. In the negative mode, a total of three compounds were putatively identified (Supplementary Figures S6, S7), two sugars and one fatty acid, respectively: D-(+)-Ribonic acid γ-lactone ([M-H + H_2_O]^−^ at *m/z* 165.04112); Inositol ([M-H]^−^ at *m/z* 179.05611) and trans-Ekode-(E)-Ib ([M-H]^−^ at *m/z* 309.20713) (Table 3). Regarding the positive ionization mode, it was possible to observe two important compounds for the summer/winter samples separation: N-Carboxyethyl γ-aminobutyric acid ([M + H]^+^ at *m/z* 176.09173) and (Z)-5,8,11-trihydroxyoctadec-9-enoic acid ([M + NH_4_]^+^ at *m/z* 348.27389) (Table 3).

**TABLE 3:** Annotated VIP responsible for season discrimination in negative and positive ionization modes.

Discrimination in the negative ESI mode						
Rt	Molecular Formula	Adduct	Theoretical *m/z*	Experimental *m/z*	Error (ppm)	Annotated compound (chemical class)
0.75	C_5_H_8_O_5_	[M-H + H_2_O]^−^	165.04112	165.03981	7.93	D-(+)-Ribonic acid γ lactone (monossacharide)
0.75	C_6_H_12_O_6_	[M-H]^−^	179.05611	179.05536	4.19	Inositol (Sugar alcohol)
12.98	C_18_H_30_O_4_	[M-H]^−^	309.20713	309.20790	−2.49	trans-Ekode-(E)-Ib (Fatty acid)
**Discrimination in the positive ESI mode**					
0.83	C_7_H_13_NO_4_	[M + H]^+^	176.09173	176.09176	-0.17	N-Carboxyethyl γ aminobutyric acid (amino acid)
10.67	C_18_H_34_O_5_	[M + NH_4_]^+^	348.27389	348.27374	-1.18	(Z)-5,8,11-trihydroxyoctadec-9-enoic acid (Fatty acid)

## 4 Discussion

It is known that *V. album* aqueous extracts have different cytotoxic activities related to their host tree (Klingbeil et al., 2013). However, a recent study conducted by our group showed that ethanolic preparations of this plant also demonstrated subspecies and host tree-antitumoral dependence (Holandino et al., 2020). Although only summer preparations were analyzed, *V. album* subsp. *abietis* was the most potent mother tincture, followed by *V. album* subsp. *album*.

The present cellular cytotoxic screen also indicated that the tumoral characteristics are related to the damage triggered by these ethanolic preparations. The metabolic cellular differences could explain the highest cytotoxic potential detected in breast cancer cells (MDA-MB-231) under incubation with different concentrations of Q-S (IC_50_ 1.48% v/v; 24 h), followed by A-S (IC_50_ 2.38% v/v; 24 h). Additionally, Pieme et al. (2014) using the same cell line, MDA-MB-231, and methanolic extract prepared with mistletoe–harvested in July at a site in Niger–detected the highest IC_50_ value (12.70 ± 0.9 mg/ml). Although these authors did not compare tumor and no tumor cells, and did not specify the host tree used, the toxicity of non-aqueous *V. album* extracts was also detected. The evaluation of the selective index (SI) is very important for determining the antitumoral potential of an herbal drug (Indrayanto et al., 2021). Our results showed that the Q-S sample did not present selective effects regarding breast cancer and fibroblastic normal cells. However, the A-S sample showed 1 < SI < 10, suggesting the need for further evaluation using different cell lines, exposure treatments, as well as other variables, to better understand the cytotoxic selectivity of this *V. album* mother tincture.

Cancer is a large variety of diseases that lead to abnormal cell proliferation with different kinetics, sites of origin and patterns of spread. It is known that the tumorigenesis process depends on the reprogramming cellular metabolism. Regarding to glucose metabolism, cancer cells are able to increase glucose uptake and aerobic glycolysis (fermentation process) even in the presence of high concentrations of oxygen, transposing the Pasteur effect (Pascale et al., 2020). The glucose is converted into pyruvate, resulting in the lactate production (Ganapathy-Kanniappan and Geschwind, 2013). This is the most common feature and well-described alteration in the energy metabolism of tumor cells. In fact, the glycolytic flux is 2–17 times greater in rat hepatomas than in normal hepatocytes (Marín-Hernández et al., 2006). The conversion of glucose to lactic acid in the presence of oxygen in cancer cells was first reported by Warburg in the 1920s and it is known as “Warburg effect” (Liberti and Locasale, 2016; Pascale et al., 2020). Since then, experimental observations of the increased glycolysis in tumors, even in the presence of oxygen, have been repeatedly verified (Bazylianska et al., 2021; Kalezic et al., 2021).

In this sense, biochemical reactions of glycolysis and their involved enzymes can be an attractive targeting for cancer therapeutic intervention. Hexokinase (HK) plays a pivotal role in tumor metabolism since it is responsible for the first step of glycolysis, in which glucose is converted into glucose-6-phosphate. This enzyme provides direct feedback inhibition that prevents the ATP cell-consumption keeping the cancer cell energy (Ganapathy-Kanniappan and Geschwind, 2013). Phosphofructokinase (PFK) catalyzes another rate-limiting step of glycolysis, and drugs able to inhibit this step are promising anticancer agents. Their reactional conversion of phosphoenolpyruvate to pyruvate leads to ATP production by glycolytic pathway. Consequently, inhibitors of this enzyme could play a significant role in the cancer treatment.

The researchers developed by our group have demonstrated the impact of harvest conditions and host tree influences on the chemical composition (Jäger et al., 2021) and biological activities (Holandino et al., 2020) of different *V. album* mother tinctures. Additionally, new important findings related to tumor metabolism were introduced for the first time by glycolytic enzyme investigation conducted in the present study. Aerobic glycolysis is characterized by increased glucose uptake and lactate production, and it is the most common feature of cancer metabolism (Bazylianska et al., 2021; Kalezic et al., 2021). Natural compounds that interact with components of glycolytic pathway can reduce the cancer cell’s ability to synthesize nucleotides, proteins, or lipids (Schulze and Yuneva, 2018). The reduction in the glucose uptake, glycolytic enzyme activities and the consequent lactate production is a very interesting effect of *V. album* MT, since this treatment probably interrupts the Warburg effect (Schulze and Harris, 2012). The energetic metabolism is on the hallmarks of cancer and can modulate the cell susceptibility to cytotoxic drugs. By assessing glycolytic enzymes activities, we evaluated the metabolic profile of the cells, and could attributed the breast cancer cells death to the inhibition of glycolytic key enzymes (e.g., HK, PFK-1, and PK) suggesting a new antitumor mechanism triggered by *V. album* mother tinctures.

Some available research reports described the anticancer effects of natural products and their relation with the glycolytic enzyme’s inhibition. Xu et al. (2017) demonstrated that a bioactive flavone (chrysin) from plant extracts promoted cell apoptosis through HK-2 suppression in hepatocellular carcinoma. Furthermore, significant inhibition in glucose uptake and lactate release was promoted by curcumin across the 4 cell lines: lung (H1299), breast (MCF-7), cervical (HeLa), and prostate (PC3). The authors conjectured that the Warburg effect inhibition was crucial for cell death, with the aid of pyruvate kinase (PKM2) down-regulation promoted by 20 μM of curcumin (Siddiqui et al., 2018).

### 4.1 *Viscum album* mother tinctures present different metabolome profiles related to host-trees and harvest season

The content of plant primary and secondary metabolites is directly influenced by several factors, such as location, seasonal variations, and in the case of parasitic plants, the host tree. Regarding these metabolites, viscotoxins are some of those that contribute to the antitumor potential of mistletoe preparations. Holandino and collaborators demonstrated that the greater content of these toxins could be related to the cytotoxic activity on the MOLT-4 and Yoshida cancer cell lines. In this sense, this study has shown the influence of the season and host tree on viscotoxin and secondary metabolite contents, as well as their influence on anticancer activity. The highest VT content in summer *V. album* mother tinctures was a pattern also observed by Urech et al. (2006) in aqueous mistletoe samples. Moreover, considering the summer samples, MT of the *V. album* subsp. *album* presented higher VT than *V. album* subsp. *abietis* and *austriacum*. Schaller et al. (1998) also demonstrated that *V. album* subsp. *austriacum* presented the smallest content of viscotoxins in aqueous extracts, compared to other subspecies. Our data suggest a relationship between the VT content and cytotoxicity, as showed by MT from *V. album* subsp. *album* that promoted the highest cell death activity (Schaller and Urech, 1996; Urech et al., 2014).

In addition to VT, our work suggested the influence of the secondary metabolites host tree-dependent in the cytotoxicity and glycolytic enzyme inhibition. So, an untargeted metabolome analysis was conducted to provide a better understanding of the chemical variability among *V. album* MT from different host trees.

Firstly, the PCA showed a clear separation, mainly concerning the botanical subspecies in both ionization modes, which could explain the differences in cytotoxic activity as follows: *V. album* subsp. *album* > *V. album* subsp. *abietis* > *V. album* subsp. *austriacum*. The PLS-DA analysis focused on host trees was able to determine the main compounds responsible for this differentiation. The negative ionization mode showed increased levels of flavanone-hexose-pentose; naringenin-pentose-hexose; 2-hydroxy-4-(6-hydroxy-5,7-dimethoxy-4-oxo-4H-chromen-3-yl) phenyl6-Ohexopyranosylhexopy-ranoside; 6′-*O*-*β*-D-apiofuranosylsweroside)4 in *V. album* subsp. *abietis* mother tinctures. These pieces of data support the previous findings that also demonstrated the presence of flavonoids in aqueous and alcoholic *V. album* extracts. Naringenin and derivatives have already been described in commercial *V. album* aqueous extracts (Iscador®) (Peñaloza et al., 2020) and alcoholic extracts (Melo et al., 2018; Jäger et al., 2021). On the other hand, the positive ionization mode showed four compounds that are important to *V. album* subsp. *abietis* discrimination: (4-(β-D-glucopyranosyloxy) phenyl) acetic acid; Syringin [5-hydroxy-6-[2-(4-hydroxy-3-methoxyphenyl) ethoxy]-2-(hydroxymethyl)-4-(3,4,5-trihydroxy-6-methyloxan-2-yl) oxyoxan-3 -yl] (E)-3-(4-hydroxy-3-methoxyphenyl) prop-2-enoate; naringenin-pentose-hexose. Regarding these compounds, syringin is a phenylpropanoid known in mistletoe species (Jäger et al., 2021), but its intensity related to *V. album* host trees is described for the first time in this work. The presence of flavonoids in MT may be associated with the reduction of the Warburg phenotype in MBA-MB-231 cells. Flavonoids can block glycolysis through regulation activity, expression, and glycolytic enzyme allosteric inhibition in many cancer cell lines (Samec et al., 2020). Furthermore, concerning the supervision by host tree in PLS-DA analysis, all eight features (*m/z*-tR) in both ionization modes were high in *V. album* subsp. *abietis* MT, what can be considered as putative biomarkers for this subspecies. However, concerning the observed *in vitro* activity, it cannot be exclusively attributed to these compounds, since they presented higher levels in *V. album* subsp. *abietis* samples in contrast to the higher activity observed in *V. album* subsp. *album* MT harvested from *Quercus* sp.

Considering the untargeted metabolome analysis focusing on subspecies influence, *V. album* subsp. *abietis* and *V. album* subsp. *album* had seventeen features with higher intensity than *V. album* subsp. *austriacum*. Among the annotated compounds responsible for sample differentiation, hyperoside is a flavonol, already described in other studies about genus *Viscum*, that corroborates the results of this study (Long et al., 2017). Additionally, hyperoside demonstrated enhanced suppression of breast cancer cell viability (MDA-MB-231 cells) in combination with paclitaxel. Notably, Long et al. (2017) highlighted that hyperoside had less cytotoxicity to normal human mammary epithelial cells (MCF-10) with increased cell apoptosis and caspase-3 activity in paclitaxel-treated breast cancer cells (Sun et al., 2020). Interestingly, naringenin-pentose-hexose appeared one more time in the PLS-DA analysis showing its importance for the subspecies and host tree discrimination. However, none of the above approaches were able to demonstrate the seasonality influence in the *V. album* MT. For this reason, an untargeted metabolome analysis focusing on season harvest was conducted. Based on this discrimination, it is possible to conclude that the positive mode was better at determining the most intense *m/z* for summer MT (176.09173, 348.27389). Ions with more intense *m/z* for winter MT were better observed in the negative ionization mode. Therefore, the MT differentiation according to the summer and winter seasons was based on compounds of the primary metabolism with *m/z* < 400. Summer samples were richer in the amino acid N-Carboxyethyl *γ*-aminobutyric acid and in the fatty acid (Z)-5,8,11-trihydroxyoctadec-9-enoic acid. On the other hand, the winter tinctures presented more sugars, such as D- (+)-Ribonic acid γ-lactone and inositol, and the fatty acid trans-Ekode-(E)-Ib.

About (Z)-5,8,11-trihydroxyoctadec-9-enoic acid, the compound most intense in summer, it is known that hydroxy fatty acids occur in appreciable amounts in the plant sphingolipids (present in the cell membrane). Several environmental parameters, especially temperature, have major effects on the physical properties of biological membranes and influence membrane fluidity, which is fundamental for plants’ survival under extreme environmental conditions (Kazaz et al., 2022). In this sense, the presence of (Z)-5,8,11-trihydroxyoctadec-9-enoic acid in higher intensity in the summer samples may be related to the stabilization of the *V. album* cell membrane in higher temperatures. Moreover, it is known that unsaturated fatty acids and a few saturated branched-chain fatty acids have been reported to exhibit anticancer activity. Dailey et al. (2011) showed that the oleic acid derivative had cytotoxic activity against human breast cancer cell line MCF-7, and its effect was dependent on the dose and branched chain of the analyzed fatty acids. The high content of omega-6 fatty acid in *Citrullus colocynthis* L. seed oil is probably related to the anticancer potential reported against colorectal cancer cell lines (Caco-2 and HCT-116) (Al-Hwaiti et al., 2021).

Another important annotated compound in positive mode (detected in higher amounts in summer samples), N-Carboxyethyl *γ*-aminobutyric acid, belongs to the class of *γ*-aminobutyric acids (GABA). This bioactive constituent is detected in fruits, vegetables, and cereals and its accumulation on plant tissues is rapidly observed as a response to biotic and abiotic stress. Other GABA’s functions are also described in plants, such as a buffering effect related to carbon and nitrogen metabolism; cytosolic pH regulation; and protection against oxidative stress (Lancien and Roberts, 2006). Additionally, the pharmacological use of exogenous GABA, derived from food was also described by Song et al. (2016). These authors showed the involvement of GABA in the proliferation inhibition of colon cancer cells (SW480 and SW620), which promoted metastasis suppression in the animal model.

Concerning the summer *V. album* mother tinctures, our results suggest the involvement of large amounts of viscotoxins, (Z)-5,8,11-trihydroxyoctadec-9-enoic acid, and N-Carboxyethyl γ-aminobutyric acid as potential candidates responsible for the cytotoxic damage induced by these ethanolic preparations in MDA-MB-231 cells. On the other hand, winter mother tincture samples presented sugars in greater intensity than the summer ones. This difference in the plant metabolism is probably related to important nutritional stocks produced by *V. album* to overcome the lower photosynthetic rate period. In addition, *V. album* ripe berries were used in the production of winter mother tinctures, and the highest sugar content in these organs probably explains the presence of many saccharides detected in their metabolome profile (Becker, 2000; Sala et al., 2012; Singh et al., 2016).

Considering the fourteen VIP annotated compounds, only syringin (Panossian et al., 1998; Deliorman and Ergun, 2000; Melo et al., 2018), naringenin derivative (Melo et al., 2018; Jäger et al., 2021), inositol (Arda et al., 2003; Peñaloza et al., 2020), D-(+)-Ribonic acid *γ*-lactone and trans-Ekode-(E)-Ib (Peñaloza et al., 2020) were previously described in *V. album* species. Among these compounds, D-(+)-Ribonic acid *γ*-lactone and trans-Ekode-(E)-Ib were already described in commercial aqueous preparations from *V. album*; however, the subspecies and host trees were not specified. So, to the best of our knowledge, this is the first time that these two compounds are annotated in *V. album*, suggesting their importance to winter harvested samples differentiation. Furthermore, nine new compounds were annotated for this vegetal species. Moreover, other compounds, in addition to these fourteen VIPs, were annotated: 49 compounds in negative and 125 in positive ionization modes (Supplementary Material-Spreadsheet metabolites). Some of these compounds comprise important chemical classes (phenolic acids, organic acids, flavonoids, terpenes), and are probably related to the cytotoxic effects triggered by *V. album* mother tinctures (Melo et al., 2018).

The present study provides basic information in which the LC-HRMS and multivariate statistical analysis can be useful in classifying *V. album* tinctures from different subspecies and host trees. Moreover, this work indicated some compounds that could serve as biomarkers for the respective subspecies according to their host tree and season harvest.

## 5 Conclusion

This study described for the first time the inhibition of the glycolytic enzymes HK, PFK-1, and PK in breast cancer cells by different subspecies of *V. album* mother tinctures. The host tree and seasonal aspects were correlated with the cytotoxicity observed in MDA-MB-231 cells. Summer MT promoted the highest cellular damage compared to winter preparations. Moreover, it was possible to identify the promising antitumoral activity of *V. album* subsp. *album*, followed by *V. album* subsp. *abietis*. Concerning the *V. album* subsp. *album*, the mother tincture prepared with mistletoe from *Quercus petraea* presented the best cytotoxic results. Higher contents of viscotoxins in the summer mother tinctures could also be responsible for this excellent bioactivity. The multivariate statistical analyses using LC-HRMS data are an efficient approach to screening and tracking metabolome differences concerning *V. album* mother tinctures, supporting their use as natural products for cancer treatment. However, further research in this area is required to expand the investigation of the biological activities of mistletoe.

## Data Availability

The original contributions presented in the study are included in the article/Supplementary Material, further inquiries can be directed to the corresponding authors. The LCMS are available with their accession IDs in the MassIVE repository: MSV000090351. [https://massive.ucsd.edu/ProteoSAFe/dataset.jsp?task=a014a6aaddd44ea5bc7740d610930098].
