# Supplementary material for: Viscum album mother tinctures: Harvest conditions and host trees influence the plant metabolome and the glycolytic pathway of breast cancer cells
Source: Front Pharmacol. 2022 Oct 31;13:1027931. doi: 10.3389/fphar.2022.1027931 (PMC9662615; doi:10.3389/fphar.2022.1027931)
Supplement: Supplementary file 1 [file DataSheet1.docx]

Supplementary Material

***Viscum album* mother tinctures: harvest conditions and host trees influence the plant metabolome and the glycolytic pathway of breast cancer cells**

**Michelle Nonato de Oliveira Melo^1,2^, Alan Clavelland Ochioni^3^, Patricia Zancan^3^, Adriana Passos Oliveira^1^, Mirio Grazi^4^, Rafael Garrett^2^, Carla Holandino^1,4*^, Stephan Baumgartner^4,5,6*^**

^1^Multidisciplinary Laboratory of Pharmaceutical Sciences, Faculty of Pharmacy, Universidade Federal do Rio de Janeiro, Rio de Janeiro, Brazil.

^2^Metabolomics Laboratory, Chemistry Institute, Universidade Federal do Rio de Janeiro, Rio de Janeiro, Brazil.

^3^Laboratório de Oncobiologia Molecular (LabOMol), Faculty of Pharmacy, Universidade Federal do Rio de Janeiro, Rio de Janeiro, Brazil.

^4^Hiscia Institute, Society for Cancer Research, Arlesheim, Switzerland.

^5^Institute of Integrative Medicine, University of Witten/Herdecke, Herdecke, Germany.

^6^Institute of Complementary and Integrative Medicine, University of Bern, Bern, Switzerland.

***Correspondence:**Corresponding Authors
st.baumgartner@vfk.ch and cholandino@gmail.com

**Tables**

Table 1. Data pre-processing parameters in MZmine 2.35

| ***Filtering*** | |
| --- | --- |
| ***Crop Filter*** |  |
| *Scans* | 0-17 min |
| *MS level* | 1 |
| *Polarity* | ­ - ou + |
| *m/z* | auto range |
| **Peak detection** | |
| ***Mass detection*** |  |
| *Scans* | 0.01 a 16.99 min |
| *MS level* | 1 |
| *Polarity* | ­ - ou + |
| *Mass detector* | *Exact mass* |
| ***Chromatogram builder*** |  |
| *Scans* |  |
| *Min. Time spam* | 0.01 |
| *Min. Hight* | 1.10E+06 |
| *m/z tolerance* | 5 ppm |
| ***Chromatogram deconvolution*** |  |
| *Algorithm* | *Wavelets* (ADAP) |
| *m/z range for MS1* | 0.01 |
| *RT range for MS1* | 0.1 |
| ***Isotopes*** | |
| ***Isotopic peaks grouper*** |  |
| *m/z tolerance* | 5 ppm |
| *RT tolerance* | 10 (% *relative*) |
| *Maximun charge* | 2 |
| *Representative isotope* | *most intense* |
| ***Normalization*** | |
| ***Retention time normalizer*** |  |
| *m/z tolerance* | 5 ppm |
| *Retention time tolerance* | 10/ % *relative* |
| *Minimum standart intensity* | 1.00E+07 |
| ***Alignment*** | |
| ***Join aligner*** |  |
| *m/z tolerance* | 5 ppm |
| *Weith for m/z* | 75 |
| *Retention time tolerance* | 10 (% *relative*) |
| ***Filtering*** | |
| ***Peak list rows filter*** |  |
| *Minimum peaks in a row* | 5 |
| *Minimum peaks in na isotope* | 2 |
| *Keep row that match all criteria* | *yes* |
| ***Gap filling*** | |
| ***Peak finder*** |  |
| *Intensity tolerance* | 10% |
| *m/z tolerance* | 5 ppm |
| *Retention time tolerance* | 10 (% *relative*) |

Table 2. Parameters for metabolite annotation in MS-Dial 4.70

| ***Data collection*** | |
| --- | --- |
| ***Mass acuracy*** |  |
| *MS1 tolerance* | 0.005 Da |
| *MS 2 tolerance* | 0.05 Da |
| ***Data collection parameters*** |  |
| *Retention time begin* | 0.5 min |
| *Retention time end* | 16 min |
| *Mass range begin* | 100 Da |
| *Mass range end* | 1000 Da |
| *MS/MS mass range begin* | 100Da |
| *MS/MS mass range end* | 1000 Da |
| ***Isotope recongnition*** |  |
| *Maximum charge number* | 1 |
| ***Multithreading*** |  |
| *Number of threads* | 4 |
| **Peak detection** | |
| ***Peak detection parameters*** |  |
| *Minimum peak height* | 5.10^5^ |
| *Mass slice width* | 0.05 Da |
| *Smoothing method* | Linear weighted moving avarage |
| *Smoothing level* | *3 scan* |
| *Minimum peak width* | *5 scan* |
| ***MS2 Deconvolution*** | |
| *Sigma window value* | 0.5 |
| *MS/MS abundance cut off* | 0 |
| *Exclude after precursor ion* | *yes* |
| *Keep the isotopic íons until* | *0.5 Da* |
| ***Identification*** | |
| *Retention time tolerance* | 100 min |
| *Accurate mass tolerance (MS1)* | 0.005 Da |
| *Accurate mass tolerance (MS2)* | 0.05 Da |
| *Identification score cut off* | 80% |
| ***Adduct*** | |
| *[M+H] ^+^* | *[M-H] ^-^* |
| *[M+Na] ^+^* | *[M-H_2_O-H] ^-^* |
| *[M+ACN+H] ^+^  or* | *[M+FA-H] ^-^* |
| *[M+H-H_2_O] ^+^* | [2M – H] ^-^ |
| ***Alignment*** | |
| *Reference file* | *QC* |
| *Retention time tolerance* | *0.1 min* |
| *MS1 tolerance* | *0.005 Da* |
| *Retention time fator* | *0.5* |
| *MS1 factor* | *0.5* |
| *Peak count filter* | *10%* |
| *N% detected in at least one group* | *30%* |
| *Sample average/blank average* | *5 fold change* |
| *Keep reference matched metabolite features* | *yes* |

Table 3. Parameters for multivariate analysis in MetaboAnalyst 5.0

| ***Data filtering*** | |
| --- | --- |
| *None (less than 5000 features)* | x |
| **Normalization overview** | |
| *Sample nornalization* | *By median* |
| *Data transformation* | *none* |
| *Data scaling* | *Auto scaling* |

**Figures**

**
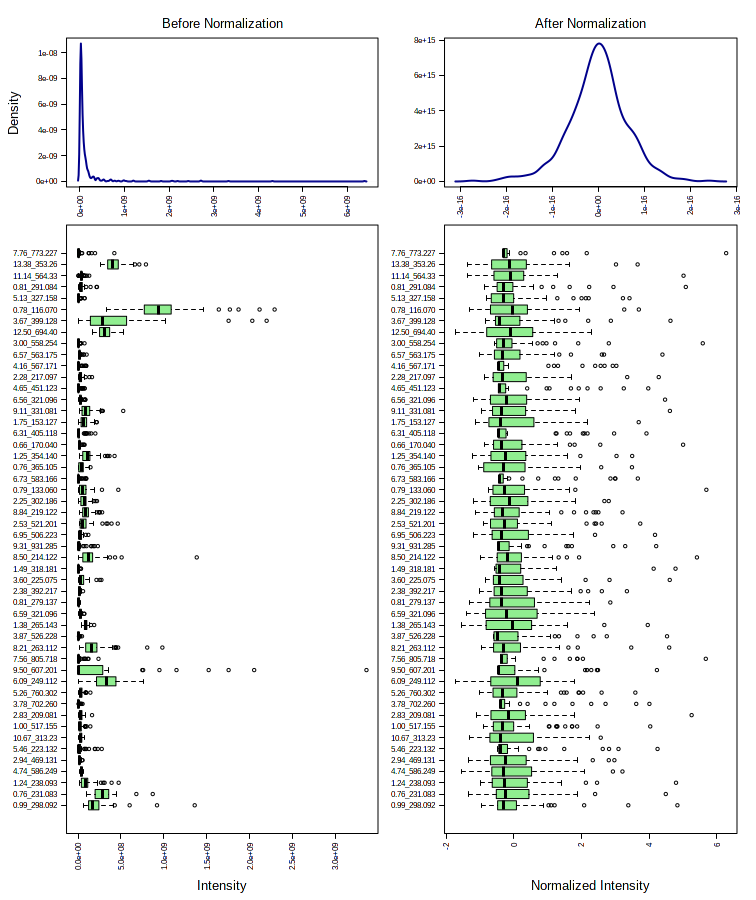
**

Figure S1. Normalization results of positive mode made on Metaboanalyst (Median normalization). The boxplots show at most 50 features/samples due to space limitation; the density plots are based on all data.


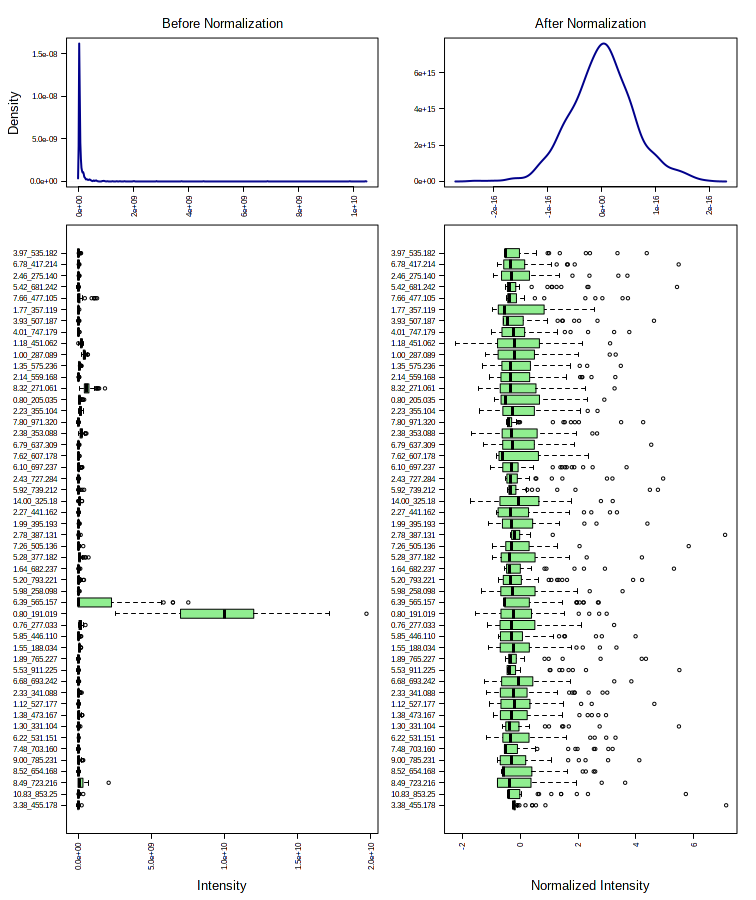


Figure S2. Normalization results of negative mode made on Metaboanalyst (Median normalization). The boxplots show at most 50 features/samples due to space limitation, the density plots are based on all data


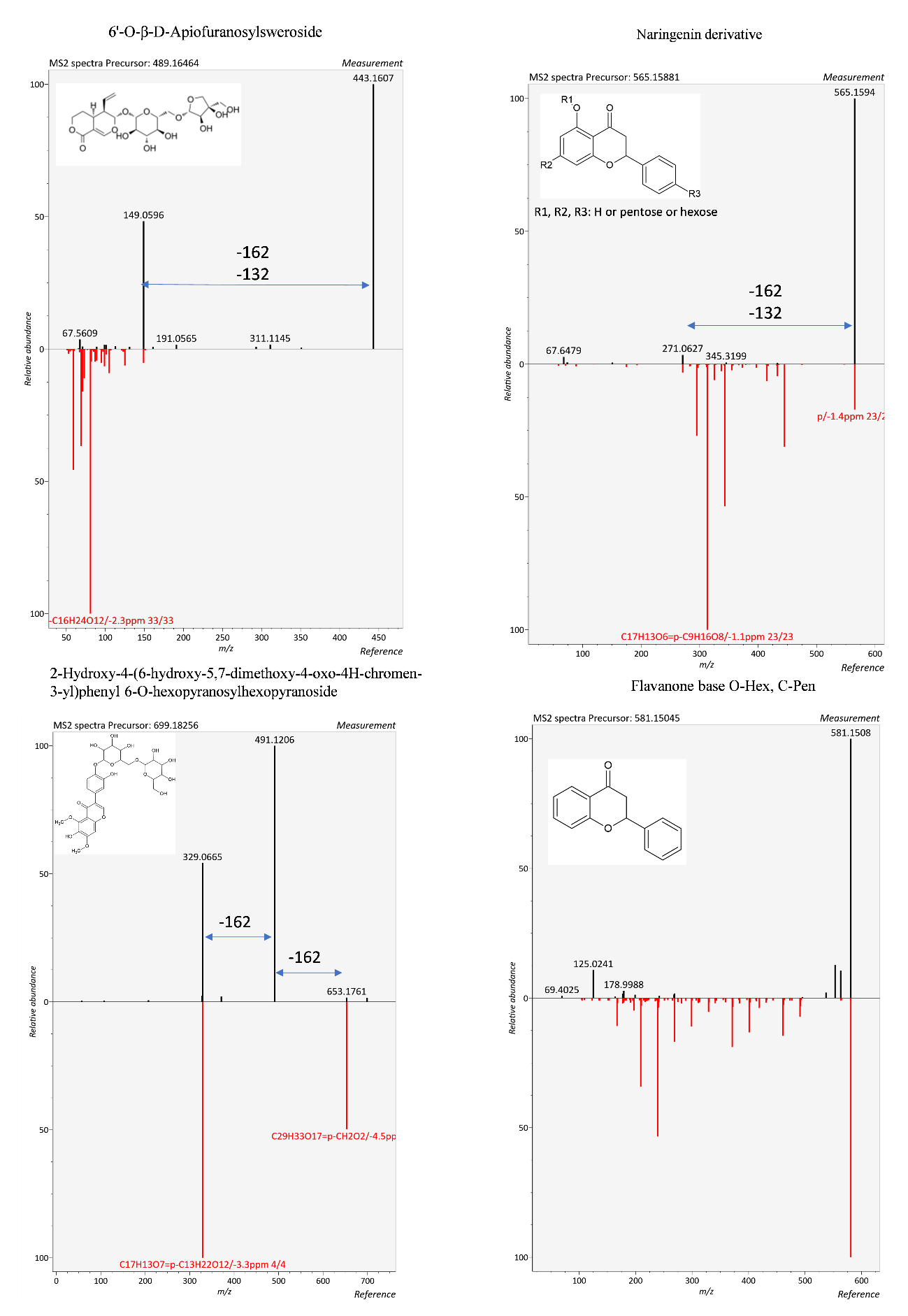


Figure S3. Experimental (black) and theoretical (red) representative MS/MS spectra in negative ESI mode, for 6'-O-β-D-Apiofuranosylsweroside, Naringenin-pentose-hexose, 2-Hydroxy-4-(6-hydroxy-5,7-dimethoxy-4-oxo-4H-chromen-3-yl) phenyl 6-O-hexopyranosylhexopyranoside, Flavanone base-hexose-pentose.


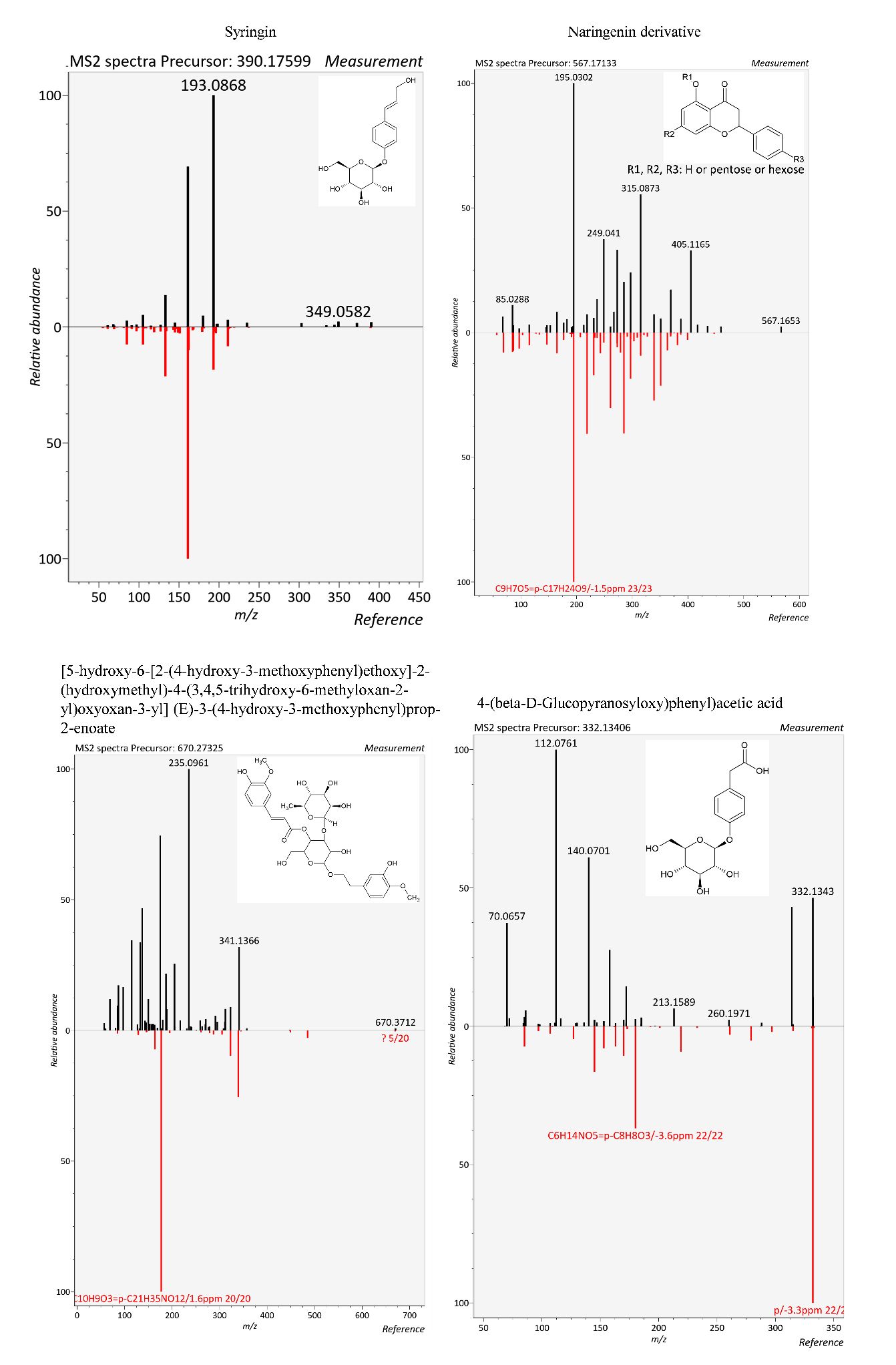


Figure S4. Experimental (black) and theoretical (red) representative MS/MS spectra in positive ESI mode, for (4-(beta-D-Glucopyranosyloxy) phenyl) acetic acid, Syringin, [5-hydroxy-6-[2-(4-hydroxy-3-methoxyphenyl) ethoxy]-2-(hydroxymethyl)-4-(3,4,5-trihydroxy-6-methyloxan-2-yl) oxyoxan-3-yl] (E)-3-(4-hydroxy-3-methoxyphenyl) prop-2-enoate, Naringenin-pentose-hexose.


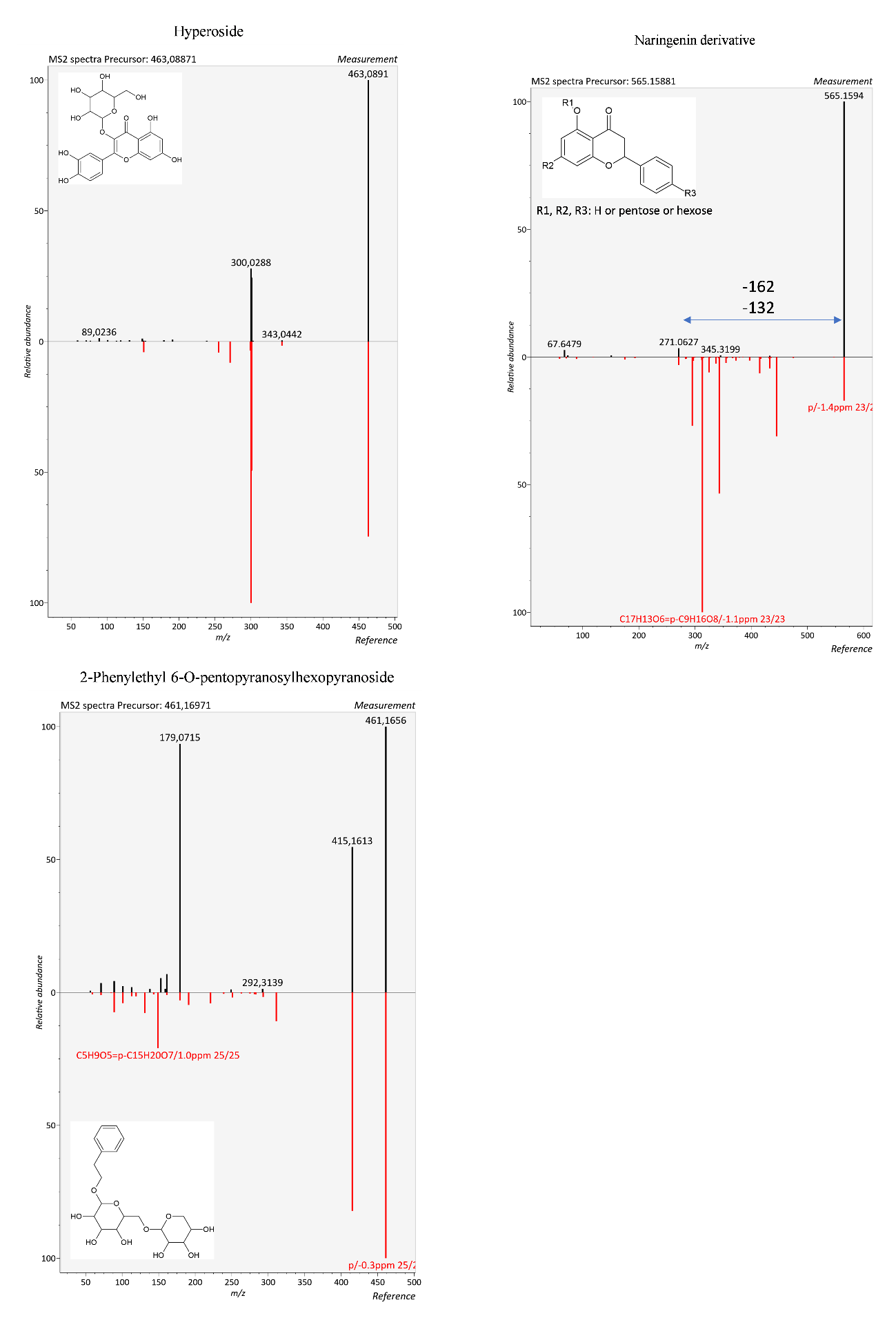


Figure S5. Experimental (black) and theoretical (red) representative MS/MS spectra in negative ESI mode, for hyperoside, naringenin derivative, 2-Phenylethyl 6-O-pentopyranosylhexopyranoside.


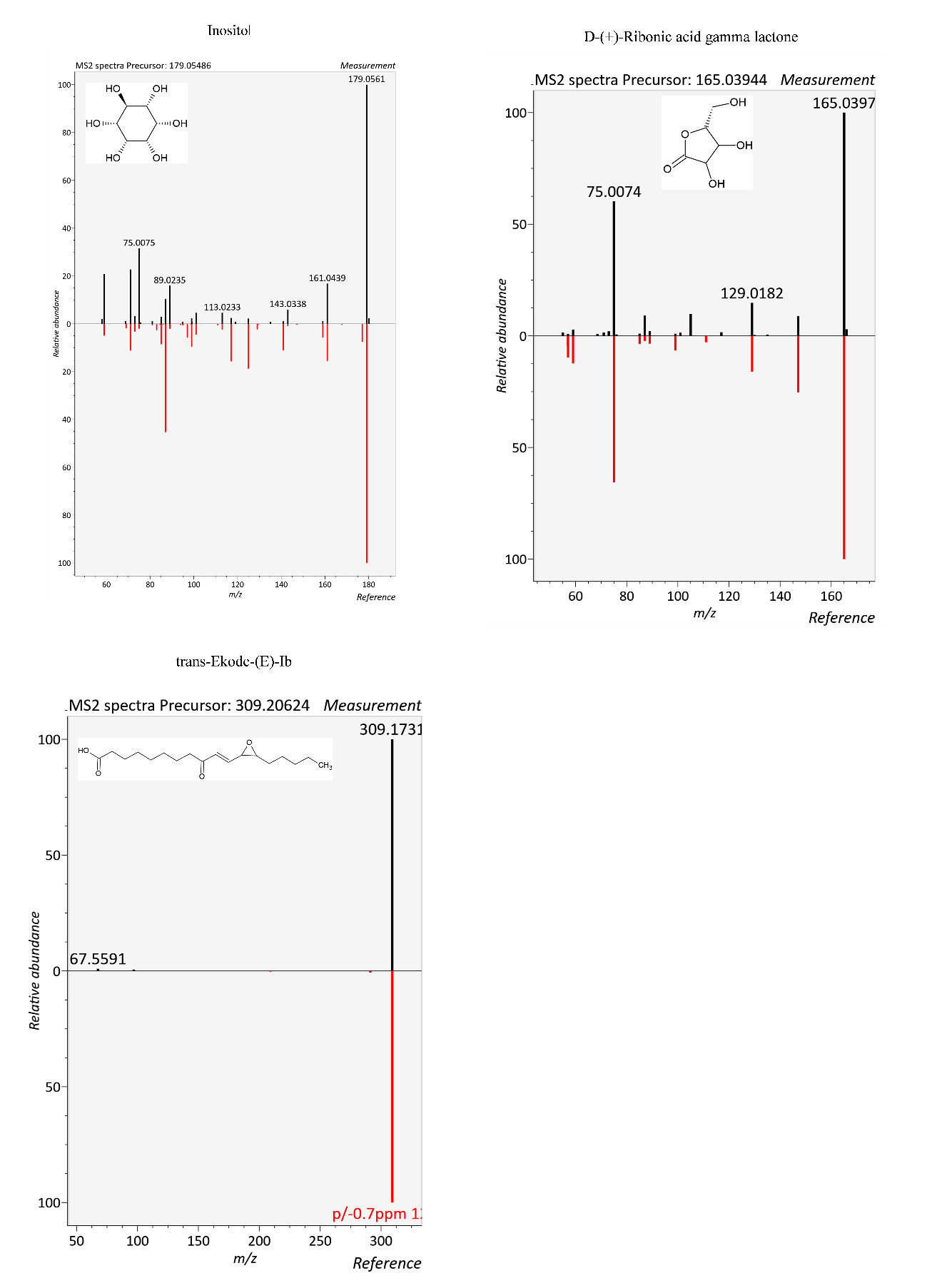


Figure S6. Experimental (black) and theoretical (red) representative MS/MS spectra in negative ESI mode, for Inositol, D-(+)-Ribonic acid gamma lactone and trans-Ekode-(E)-Ib.


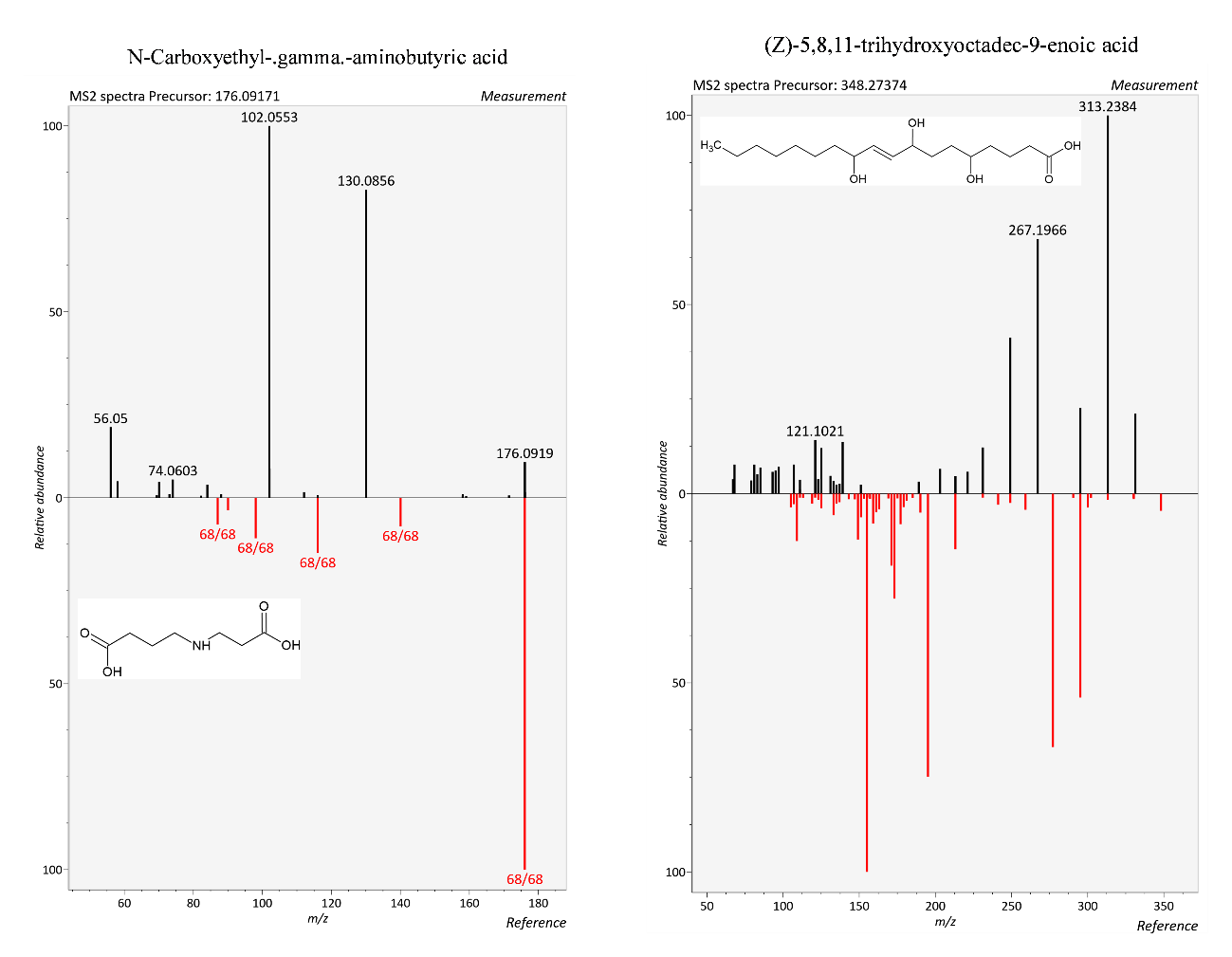


Figure S7. Experimental (black) and theoretical (red) representative MS/MS spectra in positive ESI mode, for N-Carboxyethyl-ƴ-aminobutyric acid, (Z)-5,8,11-trihydroxyoctadec-9-enoic acid.
